# Controlling Noncollinear Ferromagnetism in van der Waals Metal–Organic Magnets

**DOI:** 10.1021/jacs.4c04102

**Published:** 2024-07-02

**Authors:** Jem Pitcairn, Mario Antonio Ongkiko, Andrea Iliceto, Peter J. Speakman, Stuart Calder, Malcolm J. Cochran, Joseph A. M. Paddison, Cheng Liu, Stephen P. Argent, Andrew J. Morris, Matthew J. Cliffe

**Affiliations:** † School of Chemistry, University Park, Nottingham NG7 2RD, United Kingdom; ‡ School of Metallurgy and Materials, University of Birmingham, Birmingham B15 2TT, United Kingdom; § Neutron Scattering Division, Oak Ridge National Laboratory, Oak Ridge, Tennessee 37831, United States; ∥ Cavendish Laboratory, Department of Physics, University of Cambridge, JJ Thomson Avenue, Cambridge CB3 0HE, United Kingdom

## Abstract

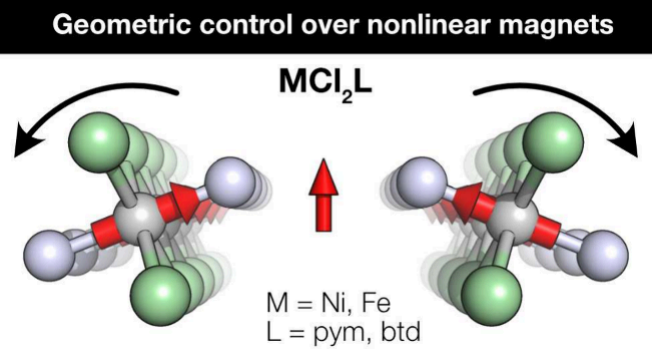

Van der Waals (vdW) magnets both allow exploration of fundamental 2D physics and offer a route toward exploiting magnetism in next generation information technology, but vdW magnets with complex, noncollinear spin textures are currently rare. We report here the syntheses, crystal structures, magnetic properties and magnetic ground states of four bulk vdW metal–organic magnets (MOMs): FeCl_2_(pym), FeCl_2_(btd), NiCl_2_(pym), and NiCl_2_(btd), pym = pyrimidine and btd = 2,1,3-benzothiadiazole. Using a combination of neutron diffraction and bulk magnetometry we show that these materials are noncollinear magnets. Although only NiCl_2_(btd) has a ferromagnetic ground state, we demonstrate that low-field hysteretic metamagnetic transitions produce states with net magnetization in zero-field and high coercivities for FeCl_2_(pym) and NiCl_2_(pym). By combining our bulk magnetic data with diffuse scattering analysis and broken-symmetry density-functional calculations, we probe the magnetic superexchange interactions, which when combined with symmetry analysis allow us to suggest design principles for future noncollinear vdW MOMs. These materials, if delaminated, would prove an interesting new family of 2D magnets.

## Introduction

The first reports of single layer ferromagnetism in the van der Waals (vdW) materials CrI_3_^1^ and Cr_2_Ge_2_Te_6_^2^ have sparked intensive efforts to realize the potential offered by two-dimensional magnetic materials for both the exploration of fundamental physics and the creation of new modalities for information technology.^3^ The most widely studied families of vdW magnets are highly symmetric and as such typically possess collinear orderings.^1,4^ The ability of more complex noncollinear magnetic order to generate new functional properties^5^ is demonstrated by one of the few exceptions to this, NiI_2_, for which there is evidence of spin-texture induced electrical polarization^6^ and predictions of skyrmion phases.^7^

Rational design of these complex, noncollinear spin textures in vdW magnets remains an open challenge. Current strategies for inducing noncollinearity includes desymmetrization through Moiré twisting, e.g. noncollinear spin textures in four-layer CrI_3_ stacks,^8^ enhancing higher-order spin–orbit derived magnetic interactions,^9^ and using lower-symmetry crystal structures. Extensive computational and theoretical searches for low-symmetry inorganic vdW materials have uncovered a handful of compounds which could host these states, such as the 1D type ordering in orthorhombic CrSBr^10,11^ and noncollinear helical edge states in candidate Weyl semimetal WTe_2_.^12−14^

Focusing solely on inorganic materials however overlooks one of the largest classes of known noncollinear magnets: coordination frameworks containing molecular ligands.^15^ The use of molecular ligands typically lowers the structural symmetry, and hence permits the interactions required for noncollinearity, such as antisymmetric Dzyaloshinskii-Moriya interactions (DMI) or canting of the local single-ion anisotropy axes. There are now a number of vdW coordination frameworks with noncollinear magnetic structures;^16−20^ however, their structural complexity also typically inhibits rational design or tuning of the magnetic interactions. Indeed, even in the highly tunable vdW metal imidazolates, MUV-1X(M) and MUV-8X(M), the deviations from collinearity cannot be readily controlled and are small.^21−23^

**Figure 1:**
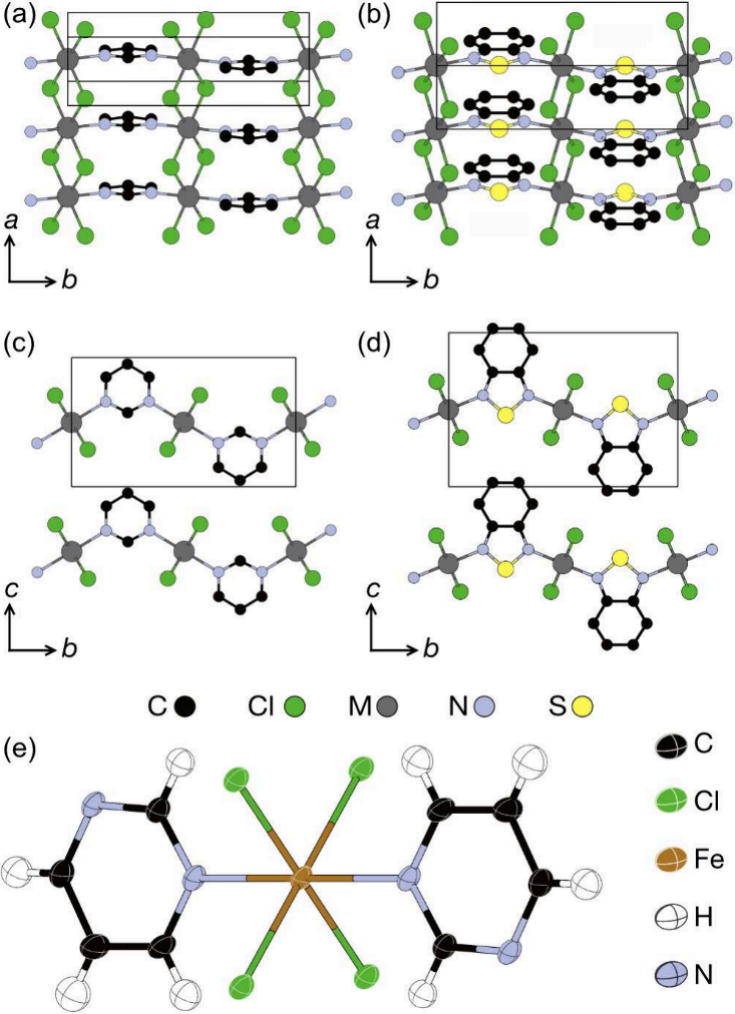
Crystal structure of MCl_2_(pym) viewed along the (a) *c*-axis and (c) a-axis and MCl_2_(btd) viewed along the (b) *c*-axis and (d) *a*-axis. The hydrogen atoms are omitted for clarity. (e) Oak Ridge Thermal Ellipsoid Plot (ORTEP) of FeCl_2_(pym) showing the coordination environment.

Metal dihalide N-heterocycles MX_2_L are a modular family of materials, in which the organic ligand, L, and metal, M, can be varied while retaining the structural connectivity: metal halide chains connected by organic ligands into 2D rectangular layers (Figure 1a,b).^24−34^ These materials are typically collinear antiferromagnets with the strongest superexchange interaction being along the MX_2_ chain. The sign of the interaction depends most strongly on the metal: Ni, Co and Fe form ferromagnetic chains^28^ and Cu and Cr form antiferromagnetic chains^26,30^ The spatial relationship between MX_2_ chains is dictated by the organic ligand: the distance between chains is determined by ligand length^25^ and the angle is controlled by the bonding geometry of the ligand (as in supramolecular cage chemistry).^31,35,36^ The controllable nature of the structure means they are an ideal family to realize targetted magnetic phases: for example both CuCl_2_(btd) and CrCl_2_(pym) have proven ripe for investigations of 1D quantum magnetism.^26,31^

In this paper we report four noncollinear bulk van der Waals magnets, MCl_2_L, where M = Ni and Fe, L = pyrimidine (pym) and 2,1,3-benzothiadiazole (btd). In each compound the noncollinearity leads to a net weak ferromagnetic moment within the layer with either a very large canting angle or large coercive field. We target these noncollinear states by connecting ferromagnetic MCl_2_ chains with strong local anisotropy^28^ using organic ligands with non 180° binding angles, thereby inducing interchain DMI interactions and ensuring the local single-ion anisotropy axes canted. We solve their structures using a combination of single-crystal X-ray diffraction (SCXRD), powder X-ray diffraction (PXRD) and powder neutron diffraction (PND), uncovering a low temperature structural phase transition in FeCl_2_(pym). Using bulk magnetic measurements and low temperature PND we determine their noncollinear magnetic ground states, showing that all four compounds possess weak ferromagnetic layers. These layers order antiferromagnetically in FeCl_2_(pym), FeCl_2_(btd) and NiCl_2_(pym), producing a fully compensated antiferromagnetic ground state, and order ferromagnetically in NiCl_2_(btd), producing a ferromagnetic ground state. Measurement of the magnetization as a function of field uncovers that all the antiferromagnetic compounds show low-field metamagnetic transitions, and both Ni compounds have very large hysteresis (μ_0_*H*_c_ > 1T, see Figure 4c,d, S13, S14) with FeCl_2_(pym) showing soft magnetic behavior, despite the antiferromagnetic ground state. Density-functional theory (DFT) calculations and diffuse scattering analysis allow us, together with symmetry arguments, to establish a hierarchy of interactions in these compounds thus rationalize their magnetic functions as arising from the competition between Heisenberg antiferromagnetic interchain exchange and spin–orbit coupling derived interactions. This allows us to suggest design rules for targetting noncollinear states in metal–organic layered magnets.

## Results

### Synthesis

Phase pure microcrystalline bulk samples of NiCl_2_(btd) and FeCl_2_(btd) were synthesized by reacting MCl_2_·nH_2_O (*n* = 4 or 6 for M = Fe or Ni, respectively) and btd without solvent in a PTFE-lined autoclave at 200 °C for 72 h. Phase pure microcrystalline bulk samples of NiCl_2_(pym) and FeCl_2_(pym) were synthesized by mixing alcoholic solutions of MCl_2_·nH_2_O and pym. We found that less polar solvents favored the formation of the monopyrimidine MCl_2_(pym) phase over the bispyrimidine MCl_2_(pym)_2_^37,38^ for both the Fe and Ni analogues. Aqueous synthesis produces the bispyrimidine phases,^37^ whereas FeCl_2_(pym) can be readily synthesized in methanol and NiCl_2_(pym) in 2:1 ethanol-diethyl ether mixtures. Single crystals suitable for X-ray diffraction of FeCl_2_(pym) were grown by the slow diffusion of pym into a methanolic solution of FeCl_2_·4H_2_O, but we were unable to grow single crystals of the other analogues.

### Crystal Structures

**Figure 2:**
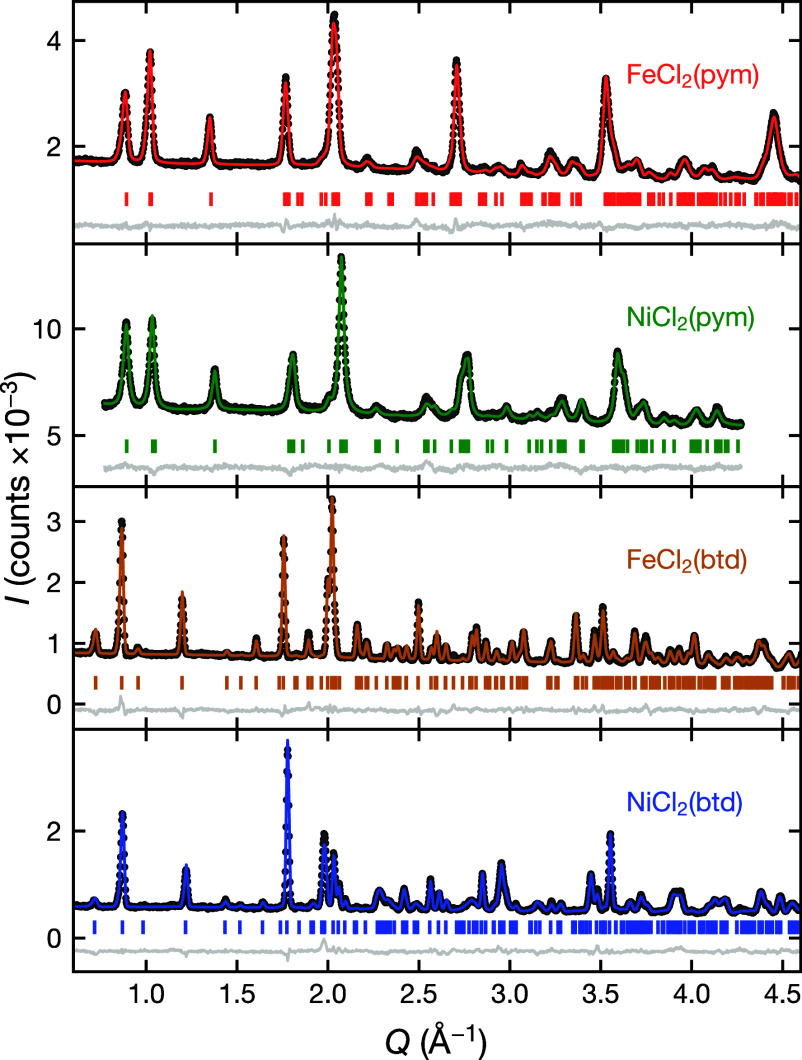
Rietveld refinement of the nuclear structures against powder neutron diffraction data. The measurement temperature for each data set is at FeCl_2_(pym), *T* = 12.5 K; FeCl_2_(btd), *T* = 5 K; NiCl_2_(pym), *T* = 2 K and NiCl_2_(btd) *T* = 2 K. For NiCl_2_(pym) the first magnetic Bragg peak (*Q* = 0.70 Å^–1^) was omitted and magnetic Bragg intensity at higher *Q* was negligible. For NiCl_2_(btd) the magnetic Bragg intensity was fixed to values determined from magnetic Rietveld refinement (see Figure 6).

Having grown diffraction-quality crystals of FeCl_2_(pym), we determined its high temperature orthorhombic structure by SCXRD at *T* = 120 K and the phase purity of the bulk microcrystalline sample was confirmed by PXRD at ambient temperature (Figure 1, S2, Table S2, S3). The low temperature monoclinic structure of FeCl_2_(pym) was determined by Rietveld refinement of PND data at *T* = 12.5K (Figure 2, Table S4). In the absence of crystals suitable for SCXRD measurements, the structure of NiCl_2_(pym) was determined by Rietveld refinement against PXRD data, using the structure of FeCl_2_(pym) as a starting model, as determined by SCXRD (Figure 1, S4). The monoclinic structures of FeCl_2_(btd) and NiCl_2_(btd) were determined by Rietveld refinement against PND data using DFT-optimized structures as a starting models (Figure 2, 1b and c). Simultaneous refinement of the nuclear and magnetic structure was undertaken for FeCl_2_(pym) and FeCl_2_(btd–d_4_) against data collected at *T* = 2K. However, the same analysis was not performed for NiCl_2_(pym) and NiCl_2_(btd–d_4_) as the signal-to-noise ratio of the magnetic Bragg peaks was not sufficient to constrain the model. We first describe the general features of the structures, before going on to describe the crystal structure and refinements in detail.

FeCl_2_(pym), FeCl_2_(btd), NiCl_2_(pym) and NiCl_2_(btd) all share a structural topology and have similar crystal structures. The M^2+^ (M = Fe, Ni) ions are coordinated by four Cl^–^ ligands and two N atoms from the pym and btd ligands, which form distorted MCl_4_N_2_ octahedra (Figure 1). The M octahedra edge-share through the Cl^–^ forming MCl_2_ chains. At ambient temperature the asymmetric units of FeCl_2_(pym) and NiCl_2_(pym) contain only one Cl^–^, so all four M– Cl bonds are equal in length, *d*_Ni–Cl_ = 2.458(2) Å and *d*_Fe–Cl_ = 2.492(5)Å (Table S2, S3). In the low temperature monoclinic phase of FeCl_2_(pym) the pyrimidine molecules are rotated about the *b*-direction, away from the *bc*-mirror plane, breaking the symmetry. This is accompanied by a small increase in the β-angle, 90° to 90.886(8)°, and a small rhombic distortion to the coordination octahedra (Figure S6). Hence, the asymmetric unit of the low temperature FeCl_2_(pym) phase contains two distinct Cl^–^, so there are two M– Cl bonds with differing lengths, *d*_Fe–Cl1_ = 2.447(5)Å, *d*_Fe–Cl2_ = 2.450(5)Å. The asymmetric units of FeCl_2_(btd) and NiCl_2_(btd) also contain two distinct Cl^–^ and two M–Cl bonds with differing lengths, *d*_Fe–Cl1_ = 2.463(8)Å, *d*_Fe–Cl2_ = 2.554(8)Å, *d*_Ni–Cl1_ = 2.441(6)Å and *d*_Ni–Cl2_ = 2.528(6)Å (Table S3). Both of the Fe compounds show a larger distortion of the M– Cl bonds than their Ni analogues, suggesting that this distortion may be driven or enhanced by a weak Jahn–Teller distortion. The MCl_2_ chains are connected into layers along the *b*-axis by μ-1,3-pym and μ-1,3-btd, but the bent ligands produce tilt angles between neighboring MCl_2_ chains of 117(1)° through pym and 132(2)° through btd (Figure 1c and d). The orientations of the pym and btd alternate up-and-down along the *b*-axis (Figure 1c and d). The corrugated vdW layers stack on top of each other along *c* (Figure 1c and d). We have chosen the space group settings so that the *a*, *b* and *c* lattice parameters correspond to the equivalent chemical directions in these four new compounds and previously reported analogues:^26^ the MCl_2_ chains lying along the *a*, the btd or pym ligands along *b* and the vdW layers along *c*.

The structure of FeCl_2_(pym) determined from SCXD data collected at *T* = 120 K and Rietveld refinement against PXRD data collected at ambient temperature shows it crystallizes in the orthorhombic space group *Pmmb* (Table S2, Figure S2). However, refinement of the nuclear structure against PND data collected at *T* = 1.5, 12.5, and 25 K reveals that FeCl_2_(pym) is in the monoclinic *P*2_1_/*m* space group at these temperatures (Figure 2, S2, Table S4). This structural phase transition can be seen in peak splitting of the peak at *Q* = 3.66 Å^–1^ and was confirmed by Rietveld refinement, where the monoclinic structure has a significantly improved fit and the cell angle refines away from 90°, β = 90.920(9)° (*R*_wp_ = 2.930, vs *R*_wp_ = 3.854 for β = 90°). We allowed the orientation of the pym to refine while keeping it as a rigid body. Our single crystal structure of FeCl_2_(pym) was then used as a starting model for Rietveld refinement of the structure of NiCl_2_(pym), initially against laboratory PXRD data (Figure S4), and then against PND data at *T* = 25 K. We found no evidence of a structural transition in NiCl_2_(pym) down to 2 K.

The structures of FeCl_2_(btd) and NiCl_2_(btd) were determined by Rietveld refinement against PND data, using the DFT-optimized structures as a starting model (Figure S3 and S5). The DFT structures were produced by geometry-optimizing models derived from the previously reported structure of CoCl_2_(btd).^32^ In our refinements, in addition to the metal and halide, we were able to refine the position of the btd ligand as a rigid body, which rotates 2.9(1)° about the *b*-axis in FeCl_2_(btd) and 1.3(1)° in NiCl_2_(btd). The structure deviation from orthorhombic is much larger in these compounds than in low temperature FeCl_2_(pym), with a markedly larger β angle, and the btd lies further from the *bc*-plane (Table S4, S5).

### Magnetometry

Having synthesized bulk samples and determined the structure of these four vdW MOMs, we sought to understand their bulk magnetic properties. The variable-temperature susceptibility, χ(*T*), for each sample was measured under field cooled (FC) and zero-field cooled (ZFC) conditions in a 0.01 T *dc* field from 2 to 300 K, and the isothermal magnetization, *M*(*H*), was measured at a range of temperatures between −5 T and 5 T for M = Fe and −14 T and 14 T for M = Ni.

#### Susceptibility

**Figure 3:**
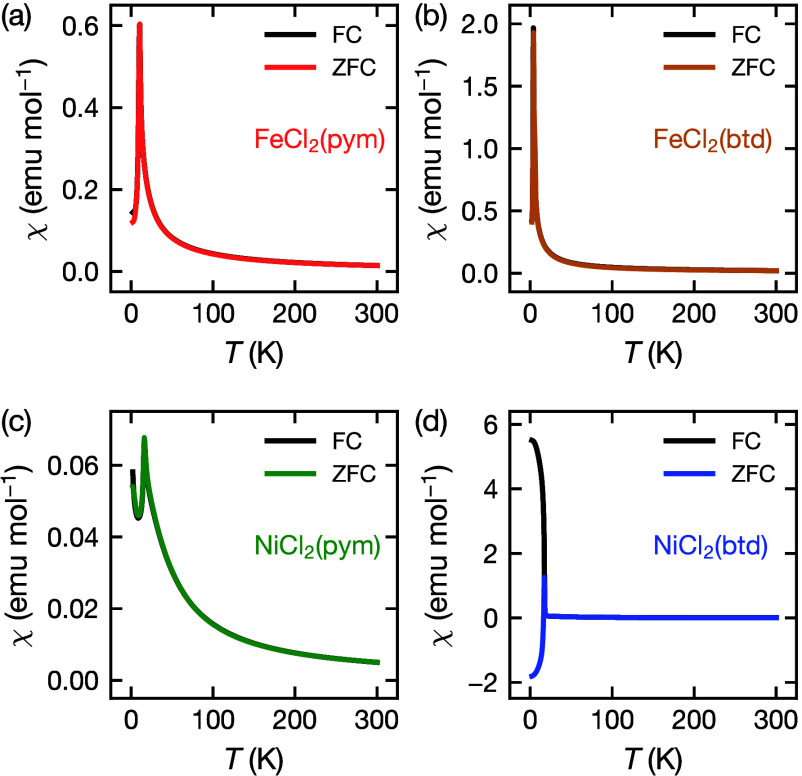
Magnetic susceptibility, χ(*T*), measurements in zero-field cooled (ZFC) and field cooled (FC) conditions from 2–300 K under a 0.01 T dc field for (a) FeCl_2_(pym), (b) FeCl_2_(btd), (c) NiCl_2_(pym) and (d) NiCl_2_(btd).

The χ(*T*) data for FeCl_2_(pym), FeCl_2_(btd) and NiCl_2_(pym) show sharp cusps at 10.5(5) K, 3.8(2) K and 15.8(7) K respectively, which are characteristic of a transition to a long-range ordered antiferromagnetic state (Figure 3). In contrast, NiCl_2_(btd) shows a bifurcation between the ZFC and FC χ(*T*) data at *T* = 17.5(5) K, indicative of ferromagnetic ordering (Figure 3d). The  data show discontinuities at these temperatures, providing further evidence of magnetic order (Figure S7d–S10d).

Fitting χ^–1^(*T*) data of FeCl_2_(pym) and FeCl_2_(btd) each at *T* >100 K using the Curie–Weiss law gave effective moments of μ_eff_ = 5.9(2) μ_B_ for FeCl_2_(pym) and μ_eff_ = 5.4(2) μ_B_ for FeCl_2_(btd), consistent with high-spin *S* = 2 Fe^2+^ and unquenched orbital angular momentum (Table 1).^39−41^ The Curie–Weiss temperatures were θ_CW_ = −2(1) K for FeCl_2_(pym) and θ_CW_ = −1(3) K for FeCl_2_(btd), indicating very small net antiferromagnetic interactions (Table 1, Figure S7c–S8c). χ^–1^(*T*) was nonlinear for FeCl_2_(btd) over the whole measured range and it was necessary to include an additional constant susceptibility term, χ_0_ = 0.008(1) emu mol^–1^, in the Curie–Weiss fit (Figure S8c).

**Table 1:** Magnetic Property Parameters Determined from Magnetic Susceptibility Measurements^a^

	FeCl_2_(pym)	FeCl_2_(btd)	NiCl_2_(pym)	NiCl_2_(btd)
*T*_*c*_ (K)	10.5(5)	3.8(2)	15.8(7)	17.5(5)
*C* (emu K mol^–1^)	4.3(1)	3.6(2)	1.47(9)	1.39(2)
θ_CW_ (K)	–2(1)	–1(3)	9(4)	22(2)
μ_eff_ (μ_B_)	5.9(2)	5.4(2)	3.43(15)	3.32(16)
*g*	2.40(8)	2.7(3)	2.42(5)	2.36(4)
*M*_*r*_ (μ_B_)	0.28(1)*	–	0.127(5)^†^	0.088(2)^†^
*H*_C_ (T)	0.2(1)*	–	1.8(1)^†^	1.0(1)^†^
*H*_*c*1_ (T)	0.2(1)*	0.04(1)*	3.8(4)^†^	–
*H*_*c*2_ (T)	1.2(2)*	0.8(1)*	6.8(2)^†^	8.3(2)^†^
γ_MH_ (deg)	16.0(6)	–	9.1(4)	6.4(3)

a *M*_*r*_ and *H*_C_ were determined from data collected at (*) 2 K and (^†^) 1.8 K.

Similarly, Curie–Weiss fitting to data measured above *T* >150 K gave an effective moment of μ_eff_ = 3.43(15) μ_B_ for NiCl_2_(pym) and μ_eff_ = 3.32(16) μ_B_ for NiCl_2_(btd) consistent with *S* = 1 Ni^2+^ (Table 1). Both Ni(II) materials had a positive Curie–Weiss temperatures indicative of net ferromagnetic exchange, θ_CW_ = 9(4) K for NiCl_2_(pym) and θ_CW_ = 22(2) K, for NiCl_2_(btd), although NiCl_2_(pym) is an antiferromagnet (Figure S9c, S10c). In all cases the presence of significant single-ion effects means that the Curie–Weiss temperature must be treated with caution.

#### Isothermal Magnetization

**Figure 4:**
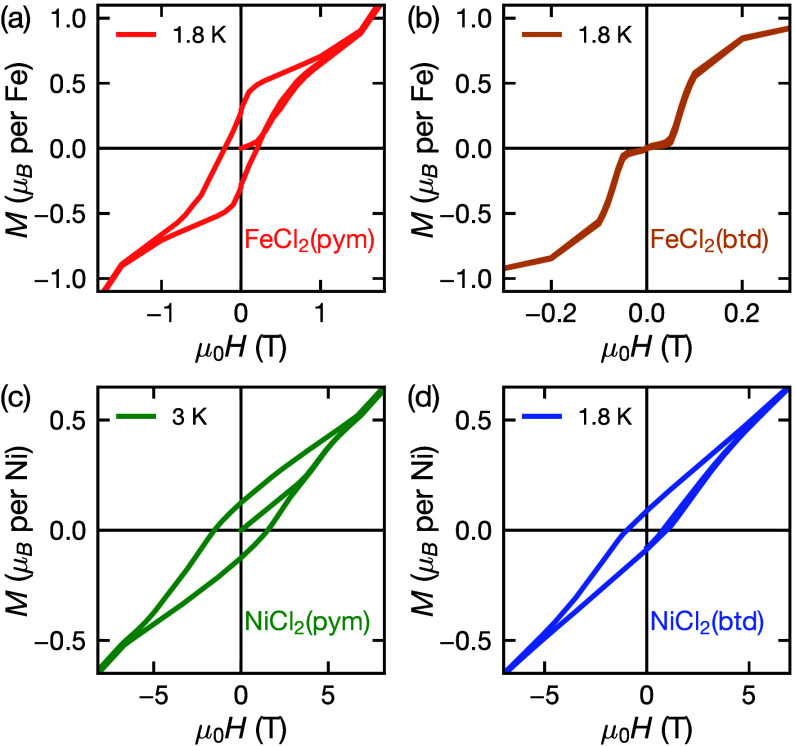
Isothermal magnetization measurements, *M*(*H*), for (a) FeCl_2_(pym) at 1.8 K between −2.6 T to 2.6 T, (b) FeCl_2_(btd) at 1.8 K between −0.3 T to 0.3T, (c) NiCl_2_(pym) at 3 K between −11 T to 11 T and (d) NiCl_2_(btd) at 1.8 K between −7 T to 7 T.

Our low temperature isothermal magnetization measurements in the ordered phases showed field-induced transitions in all samples and hysteresis in all but FeCl_2_(btd) (Figure 4). In addition to a high field transition, an additional low field metamagnetic transition occurs in antiferromagnetic FeCl_2_(pym), FeCl_2_(btd) and NiCl_2_(pym), but not in ferromagnetic NiCl_2_(btd) (Table 1, Figure S13, S14).

The isothermal magnetization measurements of NiCl_2_(pym) and FeCl_2_(pym) have analogous shapes, though with features at very different fields. On the initial sweep from zero-field *M*(*H*) increases linearly in the low field region with near constant susceptibility as expected for an antiferromagnet (Figure 4a and c). A sharp metamagnetic transition to a weak ferromagnetic state then occurs, *H*_*c*1_ = 0.2(1) T for FeCl_2_(pym) and *H*_*c*1_ = 3.8(4) T for NiCl_2_(pym). Finally a high field transition, likely to be a field polarized state, occurs at *H*_*c*2_ = 1.2(2) T for FeCl_2_(pym) and *H*_*c*2_ = 6.8(2) T for NiCl_2_(pym) (Figure S11c–e and S13c–e). There is considerable hysteresis in these transitions and the transition back to the antiferromagnetic state *H*_*c*1_ does not occur, leading to significant remnant magnetization: *M*_*r*_ = 0.28(1) μ_B_ for FeCl_2_(pym) and *M*_*r*_ = 0.127(5) μ_B_ for NiCl_2_(pym). This metamagnetic state has a considerable coercive field, *H*_C_ = 0.2(1) T for FeCl_2_(pym) and *H*_C_ = 1.8(1) T for NiCl_2_(pym) (Table 1, Figure 4a, S11a,b and S13a,b).

FeCl_2_(btd) shares the initial metamagnetic transition *H*_*c*1_ = 0.04(1) T and high field transition *H*_*c*2_ = 0.8(1) T, which are accompanied by discontinuities in  (Figure S12c–e). However, we were not able to measure any hysteresis associated with either transition. As such, there is no remnant magnetization or coercive field, and the antiferromagnetic state can be easily reached by removing the applied field.

The ferromagnetic NiCl_2_(btd) lacks the initial metamagnetic transition, but does show the high temperature field-polarized transition *H*_*c*2_ = 8 T (Figure S14c–e). It has a smaller hysteresis and remnant magnetization than the nominally antiferromagnetic NiCl_2_(pym), *M*_*r*_ = 0.088(2) μ_B_ and *H*_C_ = 1.0(1) T at *T* = 1.8 K (Figure 4d, Figure S13, S14).

**Figure 5:**
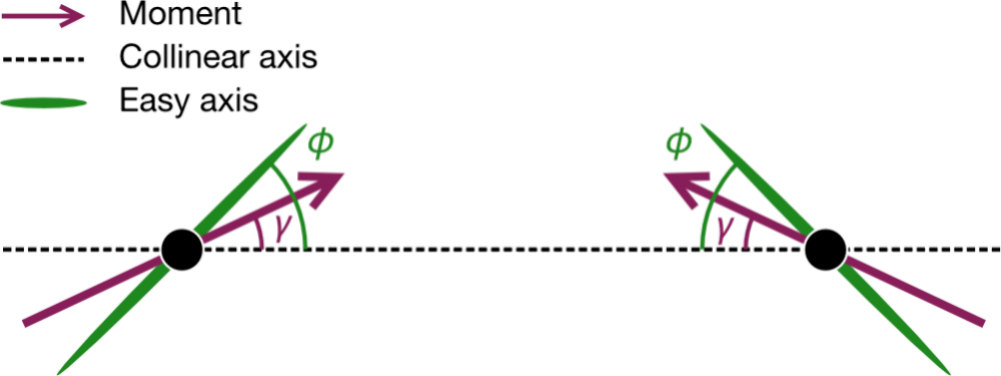
Definition of the canting angle γ and angle between the local easy-axes, ϕ and the collinear direction.

In no sample is saturation reached, strongly suggesting a noncollinear ground state. The angle between the spins and the averaged collinear axis is the canting angle, γ, which can be approximately determined from *M*(*H*) data, γ_MH_ (Figure 5). Assuming a coplanar structure and a uniaxial ferromagnetic component, the measured powder-average of the remnant magnetization can be approximated by , where *M*_⊥_ = 0.^37,42^ Hence, *M*_*r*_ can be multiplied by a factor of 3 to obtain the ferromagnetic moment along this axis, *M*_∥_ = 3*M*_*r*_. Accordingly, the canting angle γ is
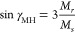
1where *M*_*s*_ is the saturation magnetization (Figure 5). The saturation magnetization was determined using the *g*-factor from Curie–Weiss analysis *M*_*s*_ = *gSμ*_B_ (Table 1). Calculating the canting angle from the directly measured *M*_*r*_ gives γ_MH_ = 9.1(4)° for NiCl_2_(pym) and 6.4(3)° for NiCl_2_(btd). However, for FeCl_2_(pym) the small coercive field means that nonlinear demagnetization, characteristic of domain structure, occurs rather than the linear dependence characteristic of continuous rotation of a canted spin. The directly measured value would therefore provide an underestimate of canting angle. To characterize just the intrinsic moment without contributions from domain structure, we determine a magnetization due to weak ferromagnetism by the extrapolation of the linear region of the hysteresis loop^43^ from *M*(μ_0_*H* = 0.1) to zero field *M*_*w*_ = 0.44(1) μ_B_, giving γ = 16.0(6)°. As there is no stable state for FeCl_2_(btd) with a net moment, we are unable to carry out equivalent analysis.

### Magnetic Diffraction

Our bulk magnetic measurements show strong evidence of long-range ordered magnetic ground states in all four compounds and so to determine the magnetic structure and correlations in their ground states, we carried out PND measurements using the HB-2A diffractometer at HFIR (ORNL). The magnetic structures were determined by refinement against data from which background and nuclear Bragg peaks were removed by subtraction of high temperature data in the paramagnetic regime. The magnetic Bragg peaks were indexed to determine the magnetic propagation vector and the possible irreducible representations (irreps) were determined using symmetry-mode analysis in the ISODISTORT software suite^44^ which are denoted below in Miller and Love’s notation.^45^ Our Rietveld refinement of nuclear structures gave us the scale factor, which we then fixed for our Rietveld refinement of the magnetic structure using each irrep against the temperature subtracted data set. Having determined the magnetic structure using temperature-subtracted data, we were then able to carry out a joint magnetic and nuclear refinement for FeCl_2_(pym) and FeCl_2_(btd) (Table S5).

**Figure 6:**
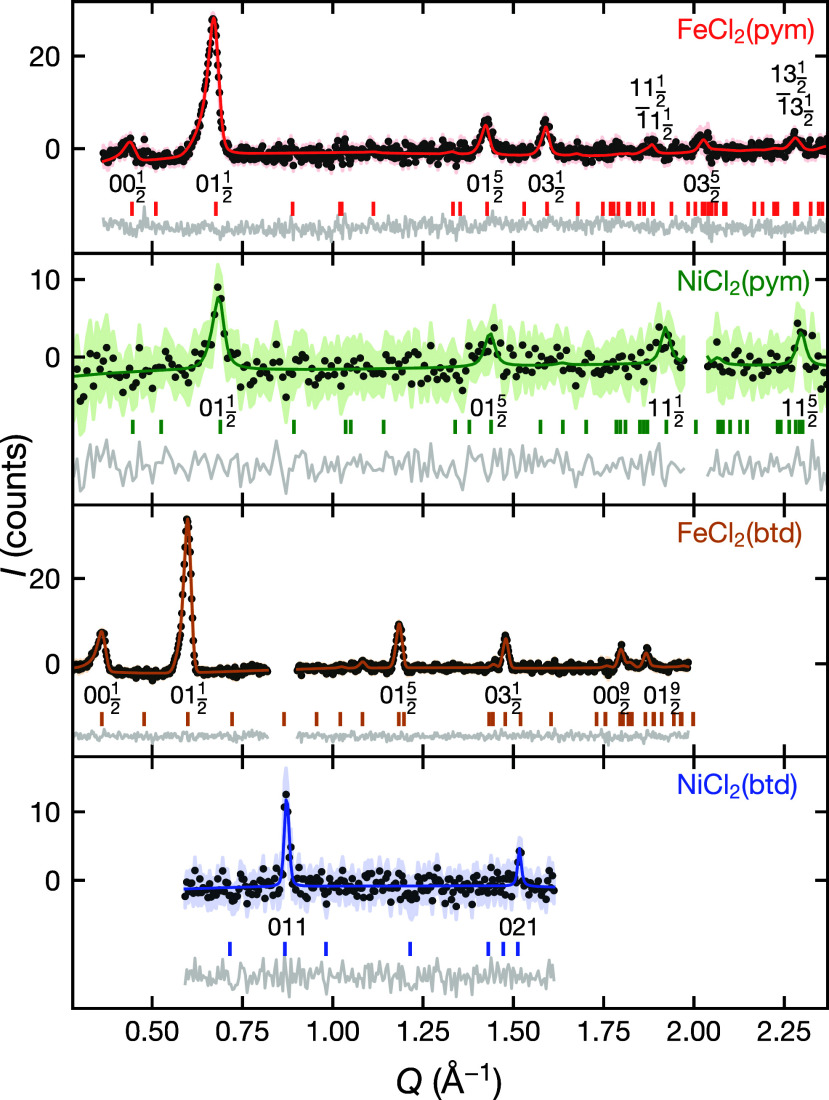
Rietveld refinement of the magnetic ground states against temperature subtracted neutron diffraction data. FeCl_2_(pym): The model was refined against the *I*_1.5 K_ – *I*_12.5 K_ data set over 0.36 < *Q* < 2.37 Å^–1^. FeCl_2_(btd): The model was refined against the *I*_1.5 K_ – *I*_5 K_ data set over 0.26 < *Q* < 1.98 Å^–1^. Data at 0.82 < *Q* < 0.09 Å^–1^ were omitted due to incomplete peak subtraction caused by thermal expansion. NiCl_2_(pym): The model was refined against the *I*_2 K_ – *I*_30 K_ data set over 0.29 < *Q* < 2.61 Å^–1^. Data at 1.97 < *Q* < 2.04 Å^–1^ were omitted due to incomplete peak subtraction caused by thermal expansion. NiCl_2_(btd): The model was refined against the *I*_1.5 K_ – *I*_30 K_ data set over 0.59 < *Q* < 1.61 Å^–1^. Data outside this range were omitted due to the absence of magnetic Bragg peaks and the presences of features arising from incomplete subtraction of structural Bragg peaks due to thermal expansion.

On cooling below *T*_*C*_ we find magnetic Bragg peaks for FeCl_2_(pym) at *T*_N_ = 10.5(5) K, FeCl_2_(btd) at *T*_N_ = 3.8(2) K, NiCl_2_(pym) at *T*_N_ = 15.8(7) K and NiCl_2_(btd) at *T*_C_ = 17.5(5) K (Figure S15). In the subtracted data sets we were able to isolate and index the magnetic Bragg peaks with propagation vectors, with the three antiferromagnets having a propagation vector  and the ferromagnetic NiCl_2_(btd) having **k** = 000, confirming its ferromagnetic ground state (Figure 6, Table 2). We identified the possible irreps in each case and carried out Rietveld refinement of the magnetic structures using every irrep for each material. We found that only one irrep was consistent with the experimental data for each material: *mB*_1_^+^ for both FeCl_2_(pym) and FeCl_2_(btd); *mZ*_1_^–^ for NiCl_2_(pym); and *m*Γ_2_^+^ for NiCl_2_(btd). We note that the observed magnetic structure for FeCl_2_(pym) would require two different magnetic irreps were the high temperature orthorhombic phase used as the parent paramagnetic phase rather than the correct monoclinic phase (ESI Sec. S1.2).

**Table 2:** Refined Magnetic Parameters from PND Analysis of the Magnetic Diffraction^a^

	FeCl_2_(pym)	FeCl_2_(btd)	NiCl_2_(pym)	NiCl_2_(btd)
Crystal system	Monoclinic	Monoclinic	Orthorhombic	Monoclinic
Magnetic space group (BNS)	*P*_*a*_2_1_/*m*	*P*_*a*_2_1_/*m*	*P*_*c*_*cca*	*P*2_1_^′^/*m*^′^
**k**-vector	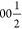	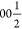	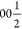	000
*M*_*x*_ (μ_B_)	–3.096(65)	–3.434(30)	0*	0^†^
*M*_*y*_ (μ_B_)	1.249(41)	1.643(22)	–2.012(70)	1.67(12)
*M*_*z*_ (μ_B_)	1.653(19)	–1.103(19)	0^†^	0^†^
*M*_0_ (μ_B_)	3.726(99)	3.866(46)	2.012(70)	1.67(12)
γ_ND_ (deg)	19.6(5)	25.2(3)	≤30	≤23
*C*_*Cl*_	FM	FM	FM	FM
*C*_*pym*/*btd*_	ncAFM	ncAFM	(nc)AFM	(nc)AFM
*C*_*vdW*_	AFM	AFM	AFM	AFM
*T* (K)	1.5	1.5	2	1.5
*R*_wp_	29.303	14.320	65.703	63.114
GOF	0.905	0.806	0.473	5.892
λ (Å)	2.41	2.41	2.41	2.41

a The ordered moment is given in the Cartesian axes: *x* = *a*_nuc._, *y* = *b*_nuc._, *z* = *c*_nuc._ × sin β. *Components prohibited by symmetry. ^†^Components fixed to zero as no magnetic intensity detected in relevant reflections.

We were able to refine the moment directions and magnitudes freely for both iron compounds, but the lower signal-to-noise due to the smaller moment for nickel meant that we were only able to put an upper limit on the noncollinear component in the ordered moment for NiCl_2_(pym) of *M* ≤ 1 μ_B_ and for NiCl_2_(btd) of *M* ≤ 0.7 μ_B_. A canting angle of γ ≤ 30° for NiCl_2_(pym) γ ≤ 23° for NiCl_2_(btd) would be therefore challenging to detect in our neutron measurements. In particular, the additional peaks that would be a signature of this noncollinearity were too small to detect. For NiCl_2_(pym) a noncollinear component along *c* is symmetry permitted and would produce Bragg intensity for 021_mag._. For NiCl_2_(btd) a noncollinear component in the *ac*-plane is symmetry permitted and could produce Bragg intensity for the 001_mag._, 020_mag._ and 021_mag._ peak positions. We therefore constrained the moment to lie along the *b*-direction in NiCl_2_(pym) and NiCl_2_(btd). These refinements produced ordered moments in good agreement with those expected: *M*_0_ = 3.726(99) μ_B_ for FeCl_2_(pym), 3.866(46) μ_B_ for FeCl_2_(btd), 2.012(70) μ_B_ for NiCl_2_(pym) and 1.67(12) μ_B_ for NiCl_2_(btd).

**Figure 7:**
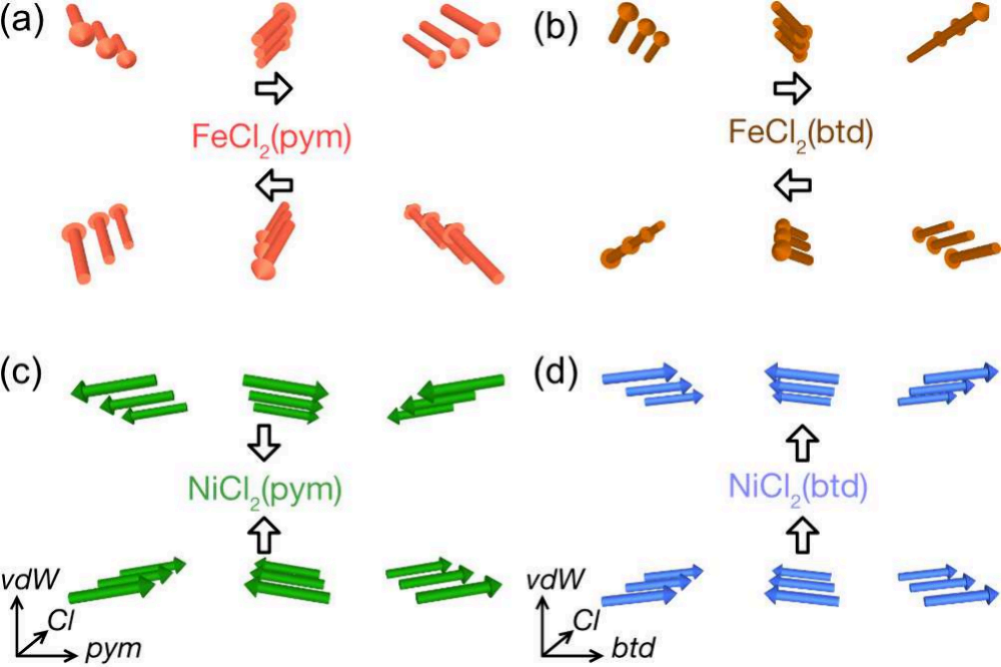
Schematic representation of the magnetic ground states of (a) FeCl_2_(pym), (b) FeCl_2_(btd), (c) NiCl_2_(pym) and (d) NiCl_2_(btd).

All four compounds have similar magnetic structures, with ferromagnetic correlations along the MCl_2_ chains and primarily antiferromagnetic correlations along the *b*-direction. The interlayer correlations are antiferromagnetic, except for NiCl_2_(btd). In the Ni compounds, the refined moments lie along the *b*-direction, though the canting is anticipated to occur along the *c* axis as this is allowed by symmetry. For FeCl_2_(pym) and FeCl_2_(btd) the moments primarily point along the *a* direction, but cant toward the *b* axis producing a net intralayer moment in this direction. This net moment is compensated for by the antiferromagnetic alignment with neighboring vdW layer (Figure 7a and b). A canting angle, γ _ND_ can be determined from the Rietveld refined structures:
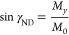
2yielding γ_ND_ = 19.6(5)° for FeCl_2_(pym) and 25.2(3)° for FeCl_2_(btd).

**Figure 8:**
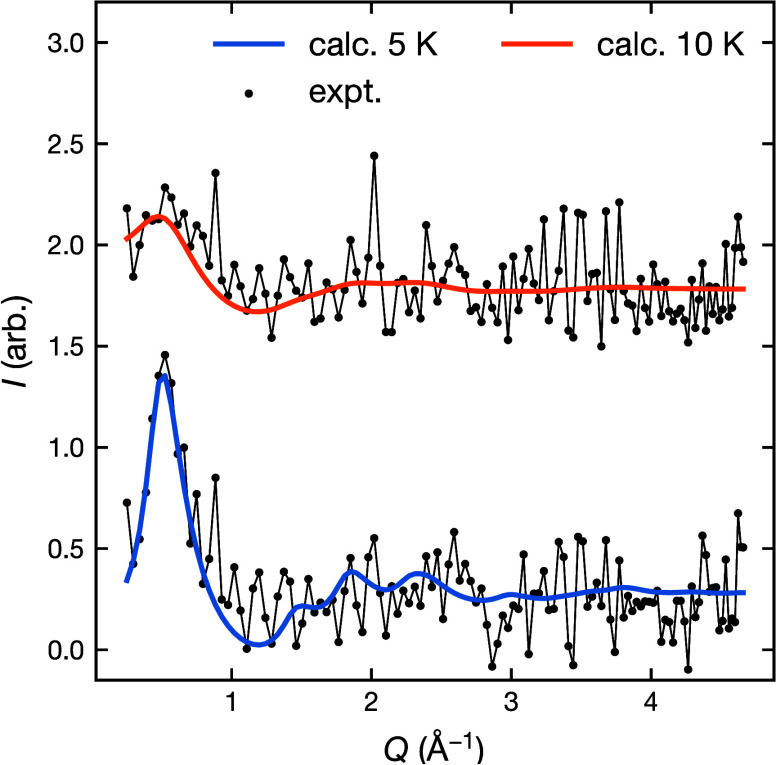
Magnetic diffuse scattering of FeCl_2_(btd) fit using an effective field model.^46^ Data obtained by temperature subtraction of data measured at 30 K.

We were also able to measure the paramagnetic diffuse scattering for FeCl_2_(btd) at 5 and 10 K by subtracting data measured at 30 K (>6*T*_N_) to account for structural scattering (Figure 8). By fitting this scattering using an effective-field model^46^ we were able to extract superexchange interactions in the Heisenberg approximation, with the Hamiltonian
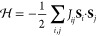
3where *J*_*ij*_ is isotropic superexchange for nearest neighbor, *J*_Cl_, and next-nearest-neighbor, *J*_btd_ interactions, and , and using a constant term to account for temperature dependent background scattering. The fit to these parameters show that *J*_Cl_ = 1.6(4) K is strongest and ferromagnetic, whereas the superexchange through the ligand, *J*_btd_ = −0.51(5) K is weaker and antiferromagnetic. Fitting with additional Heisenberg interaction terms, including *J*_vdW_, *J*_2Cl_ (i.e., the next-nearest neighbor superexchange along the FeCl_2_ chain), did not improve the quality of the fit and the refined values of these additional values were an order of magnitude smaller and zero within error. Refinement of a model with Ising spin degrees of freedom (moment directions fixed to those determined from refinement of the ground state) produced a less physical scale factor. The limited data quality means our measurements are only weakly sensitive to the single ion anisotropy and other spin–orbit derived interactions, and unfortunately prevented us from carrying out similar analysis for FeCl_2_(pym) and the Ni(II) containing compounds.

### Density-Functional Theory

To get a deeper insight into the magnetic interactions in these materials we carried out first-principles DFT calculations. For each compound we relaxed the structure, using a primitive two atom cell, and calculated the exchange energies using the broken-symmetry approach.^47^ The electronic structure and exchange energies were calculated using a collinear spin-polarized DFT Hamiltonian including a Hubbard *U* term using CASTEP,^48^ including the MBD* dispersion correction.^49^ We investigated using relativistic noncollinear DFT to prove the noncollinear magnetism found in these vdW MOMs (ESI Sec. S6.5), however these calculations were unable to provide any additional insight, due to the small energy-scales.

Geometry optimization of the primitive structures derived from single crystal X-ray diffraction data, FeCl_2_(pym) and CoCl_2_(btd)^32^ with the transition metal substituted as appropriate, produced structures consistent with those obtained by Rietveld refinement: with typical mismatches of less than 1%, and the largest deviations of 3% found for NiCl_2_(pym) (ESI Table S6). These calculations also found a small monoclinic distortion in FeCl_2_(pym), as found in our low temperature Rietveld refinement of neutron diffraction data. Examination of the electronic structure, including density of states and band structure, revealed that the inclusion of a *U* parameter was essential to avoid unphysically delocalized states. We explored a range of *U* values from *U* = 0 to 10 eV for both systems,^50−52^ determining that *U* = 2 eV for Fe and *U* = 6 eV for Ni were most appropriate. This correctly captured the experimentally observed insulating states for FeCl_2_(pym) (*E*_*g*_ = 1.30 eV), NiCl_2_(pym) (*E*_*g*_ = 2.48 eV) and NiCl_2_(btd) (*E*_*g*_ = 1.52 eV), though we found that a metallic state results for FeCl_2_(btd), likely due to strong electronic correlations on Fe(II). Our calculations for the Fe(II) compounds were very sensitive to *U* and did not reliably converge, particularly for FeCl_2_(btd), and hence we have restricted our analysis of these calculations to structural features (additional calculations in ESI S6). We note that the chain like structure appears to afford significant delocalization along the *a* direction, particularly for btd containing compounds.

We created 2 × 1 × 2 supercells from the primitive cells to allow either configurations with either FM or AFM ordering along each of the three principal directions: M-Cl-M, M-organic-M, and M interlayer. We then calculated the energies of the eight possible magnetic configurations from these supercells and fitted their magnetic superexchange interactions to the magnetic Hamiltonian (eq 3, Table 3). We found that the superexchange was very sensitive to a *U*, with too small *U* producing unphysically large exchange.

**Table 3:** Calculated Magnetic Superexchange from Collinear PBE+MBD+*U* for NiCl_2_(pym) and NiCl(btd) with *U* = 6 eV

	NiCl_2_(pym)	NiCl_2_(btd)
*J*_Cl_ (K)	29.5(4)	29.2(1)
*J*_L_ (K)	–29.0(4)	–9.7(1)
*J*_vdW_ (K)	–0.6(4)	0.0(1)

We found that NiCl_2_(pym) and NiCl_2_(btd) both had ferromagnetic *J*_Cl_ and antiferromagnetic *J*_L_ consistent with our experimental ground states, bearing in mind the nonrelativistic nature of these calculations. Surprisingly, considering the experimental ground state, we found that FeCl_2_(pym) consistently had antiferromagnetic exchange in both directions. This provides further evidence that the level of theory we are using to probe the electronic states of these Fe(II) compounds is not sufficient to accurately characterize the physics of these systems, perhaps due to the unquenched orbital moment in octahedral high spin Fe(II) and the relevance of both *t*_2*g*_ and *e*_*g*_ orbitals (which will have different localization but are treated with a single *U* in our calculations). Further details on our calculations on the iron compounds can be found in the ESI (Sec. S6). In all cases the interlayer interactions were zero within error. Focusing now on the Ni(II) compounds, we find that the interactions through pym are much more antiferromagnetic than through btd. This is consistent with the experimental Curie–Weiss temperatures, which are much less positive for NiCl_2_(pym) (θ_CW_ = +9(4) K) than for NiCl_2_(btd) (θ_CW_ = +22(2) K), though the presence of non-Heisenberg interactions prevents detailed quantitative comparison. Examination of the spin-density reveals that for both Ni(II) compounds *e*_*g*_-type d-orbitals predominate, as predicted, and the ligand spin-density primarily lies within the σ-type orbitals, for Cl^–^, pym and btd (Figure S25, S26).

## Discussion

The lattice parameters and bond lengths in these compounds follow the expected trends. The *a*- and *b*-parameters are larger for the iron compounds than the nickel, in accordance with the ionic radii, and the *a*-parameter is also slightly larger for btd containing materials than the pym compounds due to a small induced distortion in the edge-sharing MCl_2_ bridge. The *b*-parameter is significantly larger for the btd compounds than the pym compounds (≈7%) due to the μ-1,3-ligand being a five-membered ring in btd and a six-membered ring in pym. The *c*-parameter is significantly larger in the btd compounds than the pym, as the larger btd ligand separates the layers. The M– Cl– M bond angle is a key parameter in predicting *J*_Cl_,^53^ and is approximately 94° in these compounds: 93.5° for FeCl_2_(pym), 93.9° for FeCl_2_(btd), 93.6° for NiCl_2_(pym) and 94.9° for NiCl_2_(btd). We see a decrease in the angle for FeCl_2_(pym) in the high temperature phase to 91.6°, suggesting perhaps *J*_Cl_ also changes, though we find no evidence of this in our bulk magnetic data. These angles are broadly consistent with the binary halides FeCl_2_ (92.2°^54^) and NiCl_2_ (91.6°^55^), which also have nearest-neighbor ferromagnetic superexchange.^56,57^

Our neutron diffraction measurements, in combination with the bulk magnetometry, allow us to ascertain the magnetic ground state (Figure 7). We find that in all cases the moments are ferromagnetically correlated along the MCl_2_ chains, which is consistent with the only magnetic structure of Ni(II) or Fe(II) MCl_2_L analogues, NiCl_2_(4,4′-bipyridine),^27^ and as predicted by the Goodenough-Kamenari-Anderson rules.^58−60^ Our PND measurements are sensitive to the canting angle γ, which are 19.6(5)° and 25.2(3)° for FeCl_2_(pym) and FeCl_2_(btd) (Table 2). For the Ni(II) compounds the small moment size means that we were only able to measure the most intense magnetic peaks, and so our neutron measurements instead put an effective ceiling on the canting angle. These values can be directly compared with those determined from the remnant magnetization, and are broadly consistent. The size of the canting angles for FeCl_2_(pym) and FeCl_2_(btd) is very large^15^ and is comparable with some of the largest known canting angles.^37^ This noncollinear magnetic order implies the presence of multiple competing interactions (as discussed below). The magnetic neutron diffraction also clearly establishes that although in all cases there is a (potential) net moment within the layers, only NiCl_2_(btd) has ferromagnetic order, as the layers in FeCl_2_(btd), FeCl_2_(pym) and NiCl_2_(pym) are antiferromagnetically coupled. NiCl_2_(btd), FeCl_2_(pym) and NiCl_2_(pym) all show characteristics of ferromagnetism in applied field with significantly reduced *M*_*s*_ and *M*_*r*_, which, together with magnetic ground state symmetries that permits noncollinearity, strongly suggests noncollinear ferromagnetism.

The compounds reported this manuscript are unusual vdW magnets as most are inorganic and collinear including ferromagnetic CrI_3_,^1^ CrBr_3_,^61^ CrGeTe_3_,^2^ and the antiferromagnetic CrCl_3_,^62^ MPS_3_ (M = Mn, Fe, and Ni).^63−65^ While inorganic ligands provide pathways for strong superexchange, their spherical symmetry also disfavors low symmetry structures that can produce noncollinear spin textures. The potential for low symmetry organic ligands to produce spin canting in vdW MOMs is clearly demonstrated by both these compounds and the MUV-1X(M) family of MOFs.^21−23^ Many vdW magnets can be delaminated down to few layer form and this can lead to significant changes in their magnetic properties, including switching between ferro- and antiferromagnetic behavior.^1^ If MLCl_2_ can be delaminated into few- or monolayer form, we might anticipate similarities, as there appears to be a fine balance between interlayer ferromagnetism and antiferromagnetism.

FeCl_2_(pym) and NiCl_2_(pym) have the unusual combination of an antiferromagnetic ground state and magnetic hysteresis leading to remnant magnetism and a metastable zero-field ferromagnetic state. The presence of hysteresis is well-known around the metamagnetic transition, including even in canonical metamagnet FeCl_2_^66^ and in the related collinear NiCl_2_L,^25^ however the presence of metamagnetic hysteresis significant enough that the ferromagnetic state is stable without field is very rare, having been previously reported for two layered brucite cobalt hydroxides, Co_2_(OH)_3_(NO_3_) and Co_4_(OH)_2_(O_2_CC_6_H_4_CO_2_)_3_·(NH_3_)_1.5_(H_2_O)_2.5_.^67^

The coercive fields of NiCl_2_(pym) (in its ferromagnetic phase) and NiCl_2_(btd) are very large, both compared to other compounds and FeCl_2_(pym). Indeed, *H*_C_ = 1.8 T at 2 K for NiCl_2_(pym) is much larger than other vdW ferromagnets, even hard ferromagnets such as VI_3_ (*H*_C_ = 0.9T).^68−70^ The origin of this lies both with single-ion anisotropy and the magnetocrystalline anisotropy (MCA). As discussed below, Ni(II) is likely to have easy-axis, and Fe(II) easy-plane anisotropy, which suggests that the single-ion anisotropy contribution to coercivity will be larger for Ni(II). In addition, the net moment is out of plane for NiCl_2_(pym) and NiCl_2_(btd) and so will produce a larger MCA than the in plane net moment found for the Fe(II) compounds. This together likely explains the much larger coercive fields. It is notable that the related thiocyanate compound, Ni(NCS)_2_(pym)_2_ which has only M-pym-M connectivity, is also a weak ferromagnet with smaller, but still large *H*_C_ = 0.9T.^71,72^ The direct analogues Ni(NCS)_2_(pym) and Fe(NCS)_2_(pym) are antiferromagnets, as the M(NCS)_2_ chains are antiferromagnetic unlike the MCl_2_ chains.^71,72^

The large canting found in FeCl_2_(pym) parallels the compositionally similar (indeed, FeCl_2_(pym) was found to be a common impurity) but structurally distinct FeCl_2_(pym)_2_, which has only Fe-pym-Fe connections and adopts a 3D diamondoid structure.^37^ Despite the large differences in structure, these two compounds have similar magnetic properties, with *M*_*r*_ = 0.28(1)μ_B_ for FeCl_2_(pym)_2_ and *M*_*r*_ = 0.31(1)μ_B_ for FeCl_2_(pym), suggesting that care is required in the analysis of magnetic susceptibility data to ensure purity. FeCl_2_(pym) does however have an order of magnitude larger hysteresis, *H*_C_ = 0.2(1) T vs *H*_C_ = 0.015 T and a 2-fold larger magnetic ordering temperature, *T*_N_ = 10.5(5) K vs *T*_N_ = 6.5 K. The analogous diamondoid nickel compound NiCl_2_(pym)_2_ again has a slightly lower ordering temperature, *T*_N_ = 14.7(5) K, but is a collinear magnet with pseudo easy-axis anisotropy, as the easy-plane anisotropies of the NiN_4_Cl_2_ octahedra have a shared axis.^73^

The Curie–Weiss fitting and magnetic ordering temperature show interactions are stronger for NiCl_2_L than FeCl_2_L. The limitations of powder susceptibility measurements mean that we are unable to disentangle robustly the three nearest neighbor Heisenberg interactions *J*_Cl_, *J*_*L*_ and *J*_vdW_) and spin–orbit derived terms (*D*, DMI interactions) through fitting of susceptibility data. For these layered materials, considering first Heisenberg superexchange only, *T*_*c*_ will depend most critically on the strongest two superexchange interactions (≈*J*_Cl_+*J*_*L*_),^26^ and the Curie–Weiss temperature on the mean interaction (≈*J*_Cl_+*J*_*L*_+*J*_vdw_), and so *T*_*c*_ and θ are expected to be similar. The single-ion anisotropy will have a complex effect, but typically leads to a reduction in θ and increase in *T*_*c*_, explaining to some extent the observed discrepancies. Comparison with related compounds finds that both magnetic interactions tend to be stronger for Ni in both frameworks with only metal–ligand–metal connectivity^38^ and frameworks with only metal-chloride-metal connectivity.^74,75^

The expected hierarchy of interactions predicts that superexchange through the MCl_2_ chain is stronger than through the organic linker, which in turn is much stronger than between the layers, i.e., *J*_Cl_ > *J*_pym_ ≈ *J*_btd_ ≫ *J*_vdW_. This ordering was observed in our previous quantitative inelastic neutron scattering investigations of the related CrCl_2_(pym), where we found an order of magnitude separation between interactions.^26^ Fitting of magnetic diffuse scattering does confirm this picture for FeCl_2_(btd), with *J*_btd_ ≈ *J*_Cl_/3. Our DFT calculations suggest that the separation is less clear-cut for Ni(II) compounds, with *J*_pym_ ≈ *J*_Cl_ and *J*_btd_ ≈ *J*_Cl_/2.

Focusing on superexchange gives only a partial picture, as Heisenberg interactions alone will produce collinear order in a nonfrustrated magnet. Our preliminary DFT+*U* calculations including spin–orbit coupling were unable to shed significant extra light on the magnitude or directions of the key terms: however, we can produce simple guidelines from the model Hamiltonian, where we abstract away the strongest (Cl^–^) and weakest interactions (vdW) to leave a 1D metal–ligand–metal chain. The two key interactions along this chain are the single ion anisotropy and the DMI, and this model has been studied extensively for single chain magnets.^76^ In this case, as the true behavior is three-dimensional, we consider only the simplified static case. Both interactions, as they arise from spin–orbit coupling, are expected to be proportional to . The observed structures require the competition between multiple different interactions, both Heisenberg and relatistivistic.

The DMI vector, **V**, which favors a pair of spins being perpendicular to both it and each other, for NiCl_2_(pym) is normal to the pyrimidine ring by symmetry. This symmetry is broken in the monoclinic structures and instead is merely confined to the plane normal to the M–M vector. Nevertheless, as this symmetry breaking is not large, we can assume as a first approximation that the component of **V** within the ligand plane is small and that **V** lies along the plane normal. Within this approximation, the canting angle in the ordered ground state will be . In this model, the DMI would be approximately 30% the size of superexchange for Ni and roughly equal to *J* in size for Fe if the canting is driven by DMI alone. These would be large values for the DMI interaction compared to other known compounds.^77^

Both Ni(II) and Fe(II) are expected to have significant single-ion anisotropy. In both cases the local ligand field environment can be thought of as “compressed” as the four weaker-field π-donor Cl^–^ ions lie in the equatorial plane, and the σ-donor N-heterocycles are axial. The use of the term compressed is by analogy with homoleptic complexes, where compression of two bonds relative to the others will cause a similar splitting of the d orbital levels, and does not imply anything about the relative bond lengths. For d^8^ Ni(II) this leads to a strong easy-axis (Ising) type anisotropy,^42,78−81^ and for d^6^ Fe(II) this tends to produce an easy-plane (XY) type anisotropy.^78,82^ As the true symmetry is below tetragonal, there will be additional small rhombic anisotropy, *E*, neglected in this approximate treatment. There are two key parameters: the angle between the easy-axis and the M–M vector, ϕ, and the strength of the single ion anisotropy *D* (Figure 5). ϕ = 0 and 90° corresponds to collinear anisotropy and hence will produce a collinear ground state, and ϕ = 45° favors a maximally canted state, which unusually has four degenerate ordered ground states.^83^ For NiCl_2_(btd), assuming the easy-axis is coincident with the N-M-N axis gives ϕ = 22.0(5)° and for NiCl_2_(pym) this gives ϕ = 31.1(5)° due to the larger angle between the coordinating nitrogens in pym and btd. The derived canting angles are γ = 6.4(3)° for NiCl_2_(btd) and γ = 9.1(3)° for NiCl_2_(pym) (Table 1). These values rely on the validity of the assumptions made, and more accurate values could be obtained through single crystal magnetometry. Considering a Hamiltonian only containing single-ion anisotropy and Heisenberg AFM interactions for the Ni– L– Ni chain, analogous to that used in Pianet et al.,^83^ gives . Using the experimental values this implies that *D*/*J*_L_ = 0.85(4) for NiCl_2_(btd) and *D*/*J*_L_ = 0.90(3) for NiCl_2_(pym), broadly consistent with *D* observed in similar materials.^79,80,84^ The DMI and single-ion anisotropy terms will act cooperatively, and so the determined parameters thus correspond to estimates of the maxima rather than the central values. Our DFT calculations and Curie–Weiss analysis suggest that *J*_pym_ is significantly larger than *J*_btd_, which would reduce the observed canting, suggesting that the noncollinear interactions (*D* and *V*) are in fact smaller for NiCl_2_(btd).

The combination of easy-plane anisotropy and Heisenberg superexchange alone cannot produce spin canting in this model, and would instead select a unique spin direction: the intersection between the two staggered easy-planes. In these structures, the selected direction would correspond (assuming the easy-planes are oriented normal to the N-M-N axes) to spins oriented along the MCl_2_ chain normal to the plane of the organic ligand. Indeed, the spins do largely lie on this direction for both FeCl_2_(pym) and FeCl_2_(btd). The deviation of the moment direction from this axis must arise from DMI interactions, rhombic anisotropy or higher order interactions neglected in this analysis.

Our estimates of the interactions creating the noncollinear spin structures only provide an initial understanding. Future measurements will give access to more precise quantification of the underlying origin of this phenomenon: allowing us to measure the relevant higher-order interactions directly. Inelastic neutron scattering measurements, whether on single crystals or powders, would provide precise measurements of the magnetic excitations and hence *J*_Cl_, *J*_L_, *J*_vdW_ and *D*, together with indications of deviations from these terms. High field EPR and single-crystal magnetometry measurements would accurately measure *D* and *E*. Calculations using dynamical mean-field theory (DMFT) and multiconfigurational methods (e.g., CASSCF) would allow for appropriate treatment of the electron correlation and spin–orbit contributions (respectively). Our model suggests that the noncollinearity in these materials can be enhanced through further desymmetrisation of the ligand field environment: replacing the N-heterocycle with a stronger field ligand or the bridging halide with a weaker field should increase the canting angle. Equally, the geometry of the organic ligand can be used to control the noncollinearity, and a ligand that tilts the metal halide chains more would produce a larger canting angle. In particular, we note that the tilt angle for pym is 32°: if this angle can be increased to ϕ = 45°, perhaps through using more bent ligands such as 3,6-diazacarbazole or 1-alkylpyrazolo[4,3-b]pyridine, a tetrastable state would be realizable.^83^ This strategy for realizing noncollinear magnetism can be generalized to other metal–organic magnets where single ion anisotropy orientation can be anticipated.

## Conclusion

We report here the syntheses, crystal structures, bulk magnetic properties and magnetic ground states of four vdW layered MOMs: FeCl_2_(pym), FeCl_2_(btd), NiCl_2_(pym) and NiCl_2_(btd). We show they all have noncollinear ground states with large canting and net magnetic moments within each layers, and that three of these materials have significant remnant magnetization. We use density-functional calculations together with consideration of model Hamiltonians to rationalize the magnetic properties of these materials, providing a framework for the design of new noncollinear vdW MOMs.

Although we show that the choice of transition metal is the key factor determining the magnetic character of these frameworks, we also demonstrate that the organic ligand has a key influence over the resulting properties. Substituting pym for btd changes the tilt-angle between MCl_2_ chains, altering the tilt angles between chains and hence single-ion anisotropy axes, although the increased exchange in pym analogues partially counteracts this, the net result is an increased canting angle. The increased interlayer separation in btd analogues also reduces the transition magnetic fields. This suggests that the possibilities for chemical control available in MOMs will allow for tuning of spin texture, and hence potentially realizing functional properties such as magnetoelectricity^6^ or skyrmion phases.^7^

This work suggests a few clear directions forward for these materials. Our results thus far, and their limitations, suggest that a deeper understanding of the spin–orbit derived interactions will be essential to further noncollinear vdW materials design. This spans both theory, including both higher level theoretical calculations (e.g., CASSCF or dynamical mean field theory) to understand in more detail the origin of the behavior, and experimental spectroscopic characterization of the behavior, including both inelastic neutron scattering and high field EPR investigations of the magnetic excitations. The promise of these materials in bulk crystalline form also prompts us to explore whether their properties can be maintained on few- or even monolayer scale and hence toward deeper integration of these materials into 2D devices.

## Experimental Section

### Synthesis

#### FeCl_2_(pym)

The reaction of FeCl_2_·4H_2_O (3.0 g, 15 mmol; Acros Organics, ≥99%) and pyrimidine (1.2 g, 15 mmol; Sigma-Aldrich, ≥98.0%) in 50 mL methanol (MeOH) rapidly precipitates an orange-brown microcrystalline powder. The FeCl_2_(pym) product was then dried *in vacuo* giving a ca. 90% total yield. Crystals of sufficient size for X-ray diffraction studies (76 × 72 × 42 μm) were grown by vapor diffusion of pyrimidine (150 mg, 1.25 mmol; Sigma-Aldrich, ≥98.0%) into a concentrated solution of FeCl_2_ in 1 mL MeOH (20 mg, 0.16 mmol; Acros Organics, 97%). The yield was 85%. The measured (calculated) elemental composition was C, 23.03% (23.2%); H, 1.98% (1.9%); and N, 13.01% (13.4%).

#### FeCl_2_(btd)

A PTFE-lined stainless-steel autoclave was charged with FeCl_2_·4H_2_O (795 mg, 4.00 mmol; Acros Organics, ≥99%) and 2,1,3-benzothiadiazole (579 mg, 4.25 mmol; Acros Organics, 98.0%) in the solid state. The autoclave was sealed and heated solvent-free in an oven at 200 °C for 72 h. Once heating was ceased, the reaction mixture was allowed to cool gradually to room temperature. This procedure, with 2,1,3-benzothiadiazole-*d*_4_ (600 mg, 4.25 mmol; Sec.), was used to produce deuterated samples for neutron scattering studies. The yield was 93%. The measured (calculated) elemental composition was C, 25.20% (27.4%); H, 1.63% (1.5%); and N, 9.52% (10.6%).

#### NiCl_2_(pym)

The reaction of NiCl_2_·6H_2_O (173.2 mg, 0.729 mmol; Alfa Aesar, 98%) and pyrimidine (57.1 mg, 0.713 mmol; Sigma-Aldrich, ≥98.0%) in 30 mL ethanol (EtOH) rapidly precipitates a green microcrystalline powder. The NiCl_2_(pym) product was washed in 3 × 20 mL EtOH and dried *in vacuo* giving a 91% total yield. The sample used for neutron-scattering measurements was synthesized by diffusion of pyrimidine (2.0 g, 25 mmol) into a solution of NiCl_2_·6H_2_O (5.9 g, 25 mmol) in 100 mL 2:1 EtOH-diethyl ether mix. The yield was 75%. The measured (calculated) elemental composition was C, 19.63% (22.9%); H, 2.00% (1.9%); and N, 11.11% (13.4%).

#### NiCl_2_(btd)

A PTFE-lined stainless-steel autoclave was charged with NiCl_2_·6H_2_O (951 mg, 4.00 mmol; Alfa Aesar, 98%) and 2,1,3-benzothiadiazole (579 mg, 4.25 mmol; Acros Organics, 98.0%) in the solid state. The autoclave was sealed and heated solvent-free in an oven at 200 °C for 72 h. Once heating was ceased, the reaction mixture was allowed to cool gradually to room temperature. This procedure, with 2,1,3-benzothiadiazole-*d*_4_ (600 mg, 4.25 mmol; Sec.), was used to produce deuterated samples for neutron scattering studies. The yield was ca. 92%. The measured (calculated) elemental composition was C, 23.16% (27.1%); H, 3.60% (1.5%); and N, 9.22% (10.5%).

#### 2,1,3-Benzothiadiazole-*d*_4_

*o*-Phenylenediamine (2.0 g, 18.5 mmol; Sigma-Aldrich, >99%) and 20 wt % DCl/D_2_O (0.40 g, Sigma-Aldrich, ≥99.5 atom % D) were refluxed in D_2_O (50.0 g; Sigma-Aldrich, 99 atom % D) under N_2_ atmosphere for 24 h. The reaction mixture was shielded from light while being heated. After cooling, the reaction mixture was extracted with dichloromethane (3 × 50 mL). The combined organic phases were dried over MgSO_4_, filtered and concentrated *in vacuo*. The concentrated product, *o*-phenylenediamine-*d*_8_ (1.70 g, 14.6 mmol) and triethylamine (6.36 g, 58.4 mmol) were stirred to dissolution in 50 mL dichloromethane. Thionyl chloride in dichloromethane (1 M concentration, 29.2 mL) was added dropwise to the solution at 0 °C under N_2_ atmosphere in a foil wrapped flask. The solution was refluxed for 4 h under N_2_ atmosphere and concentrated *in vacuo*. 2,1,3-benzothiadiazole-*d*_4_ was purified by direct steam-distillation following addition of D_2_O acidified to pH 1 with 20 wt % DCl/D_2_O. The steam-distilled mixture was extracted with dichloromethane (3 × 50 mL) dried over MgSO_4_ and filtered. Solvent was removed *in vacuo*, affording 2,1,3-benzothiadiazole-*d*_4_ at 62% yield with 75% deuteration (1.27g, 9.05 mmol).

^1^H NMR (400 MHz, CDCl_3_, ppm, dioxane as an internal standard): δ_H_ 8.04–7.98 (m, 0.23H), 7.62–7.56 (m, 0.27H); ^13^C NMR (101 MHz, CDCl_3_, ppm): δ_C_ 154.78 (d, *J* = 5.5 Hz), 129.19 (dd, *J* = 12.5, 9.1 Hz), 121.50 (d, *J* = 11.2 Hz).

### Powder X-ray Diffraction

PXRD data were collected using a PANalytical X’Pert Pro diffractometer equipped with monochromated Cu Kα_1_ radiation (λ = 1.5406 Å). The tube voltage and current were 40 kV and 40 mA, respectively. Scans were performed from 2° to 80° on a zero background silicon crystal plate. Peak fitting, Pawley and Rietveld refinement were performed using Topas Academic v6.^85^

### Single Crystal X-ray Diffraction

A diffraction-quality single crystal of FeCl_2_(pym) was mounted on a polymer-tipped MiTeGen MicroMountTM using Fomblin (YR-1800 perfluoropolyether oil). The sample was cooled rapidly to 120 K in a stream of cold N_2_ gas, using a Oxford Cryosystems open flow cryostat. Diffraction data were collected on an Oxford Diffraction GV1000 (TitanS2 CCD area detector, mirror-monochromated Cu–Kα radiation source; λ = 1.54184 Å, ω scans). Cell parameters were refined from the observed positions of all strong reflections and absorption corrections were applied using a Gaussian numerical method with beam profile correction (CrysAlisPro). The structure was solved and refined in Olex2^86^ using SHELXT^87^ and SHELXL,^88^ respectively.

### Magnetic Susceptibility

Magnetic property measurements were first carried out on a Quantum Design MPMS superconducting quantum interference device (SQUID; School of Chemistry, University of Nottingham, **a**). Additional isothermal magnetization measurements were carried out on a Quantum Design Dynacool Physical Property Measurement system (PPMS; Cavendish Lab, University of Cambridge, **b**). Data were corrected for the diamagnetism of the sample using Pascal’s constants.^89^

### FeCl_2_(pym)

**a**: A polycrystalline sample of FeCl_2_(pym) (4.5 mg) was immobilized in eicosane (5.9 mg) and sealed in a gelatin capsule. Magnetic susceptibility measurements were performed under field cooled (FC) and zero-field cooled (ZFC) conditions in a 0.01 T *dc* field from 2 to 300 K. Isothermal magnetization measurements were performed at 2 K from 0 to 5 T to −5 to 5 T.

### FeCl_2_(btd-*d*_4_)

**a**: A polycrystalline sample of FeCl_2_(btd) (18.82 mg) was immobilized in eicosane (14.73 mg) and sealed in a gelatin capsule. Magnetic susceptibility measurements were performed under field cooled (FC) and zero-field cooled (ZFC) conditions in a 0.01 and 2 T *dc* field from 2 to 300 K. Isothermal magnetization measurements were performed at 2 K from 0 to 5 T to −5 to 5 T.

### NiCl_2_(pym)

**a**: A polycrystalline sample of NiCl_2_(pym) (12.3 mg) was immobilized in eicosane (10.7 mg) and sealed in a gelatin capsule. Magnetic susceptibility measurements were performed under field cooled (FC) and zero-field cooled (ZFC) conditions in a 0.01 T *dc* field from 2 to 300 K.

**b**: A polycrystalline sample of NiCl_2_(pym) (17.2 mg) was immobilized in cling film (7.3 mg). Isothermal magnetization measurements were performed at 1.8, 3, 4, and 8 K from 0 to 14 T to −14 to 14 T.

### NiCl_2_(btd-*d*_4_)

**a**: A polycrystalline sample of NiCl_2_(btd) (8.63 mg) was immobilized in eicosane (9.22 mg) and sealed in a gelatin capsule. Magnetic susceptibility measurements were performed under field cooled (FC) and zero-field cooled (ZFC) conditions in a 0.01 T *dc* field from 2 to 300 K.

**b**: A polycrystalline sample of NiCl_2_(btd) (22.1 mg) was immobilized in cling film (4.8 mg). Isothermal magnetization measurements were performed at 1.8, 3, 4, 8, 16, and 32 K from 0 to 14 T to −14 to 14 T.

### Powder Neutron Diffraction

Powder neutron diffraction measurements were carried out on the HB-2A neutron diffractometer at the High Flux Isotope Reactor (HFIR), Oak Ridge National Laboratory (ORNL).^90,91^ A germanium monochromator was used to select λ = 2.41 Å from the Ge(113) reflection and λ = 1.54 Å from the Ge(115) reflection. The premono, presample, and predetector collimation was open-21‘-12‘. A pyrolytic graphite (PG) filter was placed before the sample to remove higher order reflections for λ = 2.41 Å. The samples were contained in a 6 mm diameter vanadium can and cooled in a liquid ^4^He cryostat with an in situ 3-sample changer stick in the temperature range 1.5 to 300 K. The diffraction patterns were collected by scanning a 120° bank of 44 ^3^He detectors in 0.05° steps to give 2θ coverage from 5° to 130°. The magnetic structures were determined by refinement against data from which background and nuclear Bragg peaks were removed by subtraction of data collected at *T* > *T*_N_ from those collected at *T* = 1.5 or 2 K. The magnetic Bragg peaks were indexed to determine the magnetic propagation vector and then the allowed magnetic irreducible representations were determined using symmetry-mode analysis on the ISODISTORT software.^44^ Using the scale factor determined from Rietveld refinement of the nuclear structure, and peak parameters determined from Pawley refinement of the nuclear structure, the direction and magnitude of the ordered moment for the subtracted data set were refined using TOPAS-ACADEMIC 6.0.^85^

### FeCl_2_(pym)

Diffraction patterns were collected at *T* = 1.5, 12.5, and 25 K with λ = 2.41 Å for 4 h, 4h and 2h, respectively, and at *T* = 1.5 and 12.5 K with λ = 1.54 Å for 4 h each. Additional patterns were collected for 1 h at λ = 2.41 Å at intermediate temperature points *T* = 5, 6, 7, 8, 9, 9.5, 10, 10.5, 11, 12, 13, 14, and 15 K.

### FeCl_2_(btd-*d*_4_)

Diffraction patterns were collected at *T* = 1.5, 5, 10, and 30 K with λ = 2.41 Å for 3 h each. Additional data were collected with λ = 2.41 Å at *Q* = 0.60 Å^––1^ from *T* = 1.5 to 10 K in 0.5 K increments.

### NiCl_2_(pym)

Diffraction patterns were collected at *T* = 2 and 30 K with λ = 2.41 Å for 3 h each. Additional data were collected with λ = 2.41 Å at *Q* = 0.68 Å^––1^ from *T* = 2 to 30 K in 3 K increments.

### NiCl_2_(btd-*d*_4_)

Diffraction patterns were collected at *T* = 1.5 and 30 K with λ = 2.41 Å for 3 h each. Additional data were collected with λ = 2.41 Å at *Q* = 0.87 Å^––1^ from *T* = 2 to 26 K in 1 K increments.

### DFT Calculations

Calculations were carried out using the plane-wave density-functional theory code CASTEP version 23.1.^48^ The PBE general gradient approximation exchange-correlation functional was used^92^ with norm-conserving pseudopotentials from the built-in NCP19 library. Calculated exchange interactions were robust to changes in plane-wave cutoff energy for the basis set. van der Waals forces between each layer were described using the many-body semiemprical dispersion correction MBD*.^49^ An effective on-site interaction parameter, *U*_eff_ = *U* – *J*, was necessary to impose a strong localization on the Fe and Ni d-states, where *U* is the on-site Coulomb term and *J* is the site exchange term. *U*eff is applied as a correction to the total energy of the system,
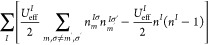
4where *n*_*m*_^*Iσ*^ are localized orbital occupation numbers with atomic site index *I*, state index *m*, and spin σ.^93^*n*_*m*_^*Iσ*^ is calculated as the projection of occupied Kohn–Sham DFT orbitals on a localized basis set. *U*_eff_ is set in CASTEP as a parameter that applies to all the orbitals within a given subshell (e.g., d-subshell).

A Monkhorst–Pack grid of *k*-points was used to integrate the Brillouin zone, with a *k*-point spacing finer than 2π × 0.03 Å^–1^ and with a plane-wave basis comprising plane-waves with energy up to 1500 eV. During the electronic minimization process a Gaussian smearing scheme was used with a smearing width of 0.2 eV. The geometry was optimized until forces were less than 0.05 eV/Å. The OptaDOS code in combination with the Matador high-throughput environment were used to generate the electronic band structures and density of states.^94−97^ The C2X visualization tool was used to obtain the spin-density representations.^98^

## Data Availability

Additional research data for this article may be accessed at no charge and under CC-BY license at the University of Nottingham Research Data Management Repository (DOI: 10.17639/nott.7395).

## Supporting Information Available

Magnetic crystal structural information files (ZIP)
Discussion on the susceptibility of FeCl_2_(btd) and magnetic symmetry analysis of FeCl_2_(pym); additional powder X-ray and neutron diffraction data, refined lattice parameters, isothermal magnetization measurements, magnetic susceptibility analysis, refined magnetic ground states, UV–vis spectroscopy, additional details of DFT calculations (PDF)
