# Supplementary material for: Controlling Noncollinear Ferromagnetism in van der Waals Metal–Organic Magnets
Source: J Am Chem Soc. 2024 Jul 2;146(28):19146–59. doi: 10.1021/jacs.4c04102 (PMC11258693; doi:10.1021/jacs.4c04102)
Supplement: Supplementary file 2 — ja4c04102_si_002.pdf [file ja4c04102_si_002.pdf]

Controlling non-collinear ferromagnetism in van der Waals metal-organic magnets

Jem Pitcairn[a], Mario Antonio Ongkiko[b], Andrea Iliceto[b], Peter Speakman[a], Stuart Calder[c], Malcolm J. Cochran[c], Joseph A. M. Paddison[c], Cheng Liu[d], Stephen P. Argent[a], Andrew J. Morris[b], and Matthew J. Cliffe *[a]

[a]School of Chemistry, University Park, Nottingham, NG7 2RD, United Kingdom
[b]School of Metallurgy and Materials, University of Birmingham, Elms Rd, Edgbaston, Birmingham B15 2TT, United Kingdom
[c]Neutron Scattering Division, Oak Ridge National Laboratory, Oak Ridge, Tennessee 37831, USA
[d]Cavendish Laboratory, Department of Physics, University of Cambridge, JJ Thomson Avenue, Cambridge CB3 0HE, United Kingdom

July 1, 2024

Contents

List of Figures **2**

List of Tables **2**

S1 Extended discussion **3**
S1.1 Temperature independent paramagnetism in $FeCl_2$(btd) . . . 3
S1.2 Magnetic symmetry analysis of $FeCl_2$(pym) . . . 3

S2 Rietveld refinements and fit parameters **4**

S3 Magnetic susceptibility **11**

S4 Powder neutron diffraction **19**

S5 UV-Vis spectroscopy **22**

S6 Density functional theory **23**
S6.1 DFT geometry optimizations . . . 23
S6.2 DFT calculated superexchange . . . 24
S6.3 Band-structure and density of states . . . 24
S6.4 Spin density . . . 26
S6.5 Visualising Kohn-Sham orbitals . . . 29
S6.6 Non-collinear relativistic DFT . . . 32

*matthew.cliffe@nottingham.ac.uk

List of Figures

S1 Magnetic Rietveld fit metric for $FeCl_2$(pym). . . . 4
S2 Structural Rietveld refinements for $FeCl_2$(pym). . . . 5
S3 Structural Rietveld refinements for $FeCl_2$(btd−d_4). . . . 6
S4 Structural Rietveld refinements for $NiCl_2$(pym). . . . 7
S5 Structural Rietveld refinements for $NiCl_2$(btd−d_4). . . . 8
S6 High and low temperature structures of $FeCl_2$(pym). . . . 8
S7 Temperature dependent susceptibility of $FeCl_2$(pym). . . . 11
S8 Temperature dependent susceptibility of $FeCl_2$(btd). . . . 12
S9 Temperature dependent susceptibility of $NiCl_2$(pym). . . . 13
S10 Temperature dependent susceptibility of $NiCl_2$(btd). . . . 14
S11 Field dependent magnetisation of $FeCl_2$(pym). . . . 15
S12 Field dependent magnetisation of $FeCl_2$(btd). . . . 16
S13 Field dependent magnetisation of $NiCl_2$(pym). . . . 17
S14 Field dependent magnetisation of $NiCl_2$(btd). . . . 18
S15 Temperature dependent PND. . . . 19
S16 Magnetic ground states of $FeCl_2$L. . . . 20
S17 Magnetic ground states of $NiCl_2$L. . . . 21
S18 UV-Vis spectra of MCl_2L. . . . 22
S19 Band structure and DOS of $FeCl_2$(pym). . . . 24
S20 Band structure and DOS of $FeCl_2$(btd–d_4). . . . 25
S21 Band structure and DOS of $NiCl_2$(pym). . . . 25
S22 Band structure and DOS of $NiCl_2$(btd–d_4). . . . 26
S23 Spin density isosurfaces of $FeCl_2$(pym). . . . 26
S24 Spin density isosurfaces of $FeCl_2$(btd–d_4). . . . 27
S25 Spin density isosurfaces of $NiCl_2$(pym). . . . 28
S26 Spin density isosurfaces of $NiCl_2$(btd–d_4). . . . 28
S27 HOMO and LUMO of $FeCl_2$(pym). . . . 29
S28 Orbitals of $FeCl_2$(btd–d_4) at the Fermi energy and around the sub-Fermi gap. . . . 30
S29 HOMO and LUMO of $NiCl_2$(pym). . . . 31
S30 HOMO and LUMO of $NiCl_2$(btd–d_4). . . . 31
S31 Non-collinear spin directions on ground state MCl_2L. . . . 33

List of Tables

S1 Irreps for $FeCl_2$(pym) magnetic ground state. . . . 4
S2 Crystal data for $FeCl_2$(pym). . . . 9
S3 Refined lattice parameters from powder XRD. . . . 9
S4 Refined lattice parameters from nuclear PND. . . . 10
S5 Refined lattice parameters from nuclear and magnetic PND. . . . 10
S6 Calculated lattice parameters from collinear PBE+U+MBD* DFT. . . . 23
S7 Calculated magnetic superexchange from collinear PBE+U+MBD*. . . . 24
S8 Calculated lattice parameters from non-collinear LDA DFT. . . . 32

S1 Extended discussion

S1.1 Temperature independent paramagnetism in $FeCl_2$(btd)

Unlike the other materials discussed in this thesis, for $FeCl_2$(btd) it was necessary to include a term for temperature independent paramagnetism to perform a Curie-Weiss analysis (Fig. S8). While this may arise from the intrinsic properties of $FeCl_2$(btd), an impurity being the source of this signal cannot be ruled out. A likely candidate is iron metal, a soft ferromagnet up to $T > 1000$ K,[1] which would produce a large, positive, near constant susceptibility at $2 < T < 300$ K. The temperature independent signal could be accounted for by as little as 10^{-4} mol% of Fe atoms in the form of iron metal, were they to saturate under the applied field.[2] If the temperature independent paramagnetism is a property of phase pure $FeCl_2$(btd), it could arise from van Vleck paramagnetism or from the delocalisation of π electrons over a $\pi - d$ conjugated network and/or π-overlap between btd ligands, producing a metallic band structure.[3,4] Indeed, the broad absorbance ion the UV-Visible spectrum of $FeCl_2$(btd) may indicate greater charge delocalisation between metals and ligands than the other compounds (Fig. S18).

S1.2 Magnetic symmetry analysis of $FeCl_2$(pym)

To confirm that the ground state symmetry of $FeCl_2$(pym) is monoclinic, extensive symmetry analysis was undertaken. We also investigated the magnetic ground state of $FeCl_2$(pym) to explore whether it was consistent with the room temperature orthorhombic structure or low temperature monoclinic structure. This was done by lowering the symmetry of the crystallographic parent cell to $P1$, removing all symmetry relations between the two symmetry distinct Fe sites. Using the propagation vector, $\mathbf{k} = 00\frac{1}{2}$, there was one possible irrep, mZ_1. Rietveld refinement in this subgroup fit the data well, confirming that the propagation vector was correct. To explore whether whether a model with higher symmetry was also able to fit the data the moments on the two metal sites were constrained in the following manner:

$$\mathbf{M}_{xi} + \mathbf{M}_{yi} + \mathbf{M}_{zi} = u\mathbf{M}_{xj} + v\mathbf{M}_{yj} + w\mathbf{M}_{zj},$$

where u, v, w can take the values -1, 0, 1.

Refinements were performed for all 27 permutations to determine the relation of best fit (Fig. S1). The results of this analysis are displayed as a three panel colour plot, where darker tiles represent better fits and lighter represent worse fits (Fig. S1). It is clear from these data that the best fit is (−1,1,−1) with $R_{\text{wp}} = 29.3$, the bottom right tile of the left hand panel. This symmetry relationship corresponds to the linear combination of the irreps $mZ_2^- + mZ_4^-$ in the high temperature $Pmma$ crystallographic phase (Tab. S1). This lowers the magnetic space group symmetry from $Pmma1'$ to P_a2_1/m and changes the crystal system from orthorhombic to monoclinic. The data are also fit, though not quite as well, by (−1,1,0) with $R_{wp} = 29.6$, in the centre right tile of the left hand panel. However, none of the possible maximal or non-maximal symmetry subgroups possess this set of relations, so this configuration was ruled out. Every other relation failed to fit at least one magnetic Bragg peak. These can be fit better still in the low temperature $P2_1/m$ monoclinic structure using the single irrep mB_1^+ with $R_{\text{wp}} = 28.7$. This analysis thus confirms that the magnetic symmetry itself is monoclinic, even without the observed structural distortion.

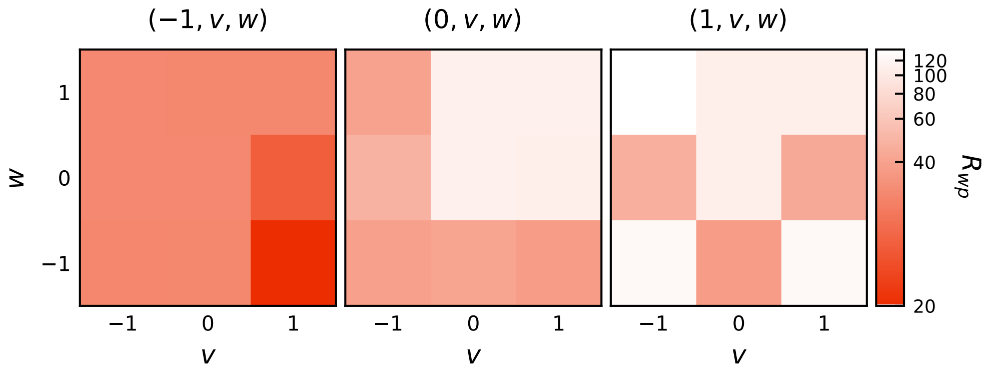

Figure S1: The R_{wp} from Rietveld refinement of the **Fe-pym** ground state with relation (u,v,w) between each the symmetry distinct magnetic Fe sites.

Table S1: The possible single irreps and the best multiple irrep describing the magnetic ground state of **Fe-pym** determined by symmetry-mode analysis[5] and the symmetry relations between the Fe sites.

Irrep(s)	u	v	w	Mag. space group	R_{wp}
mZ_1^-	0	−1	1	$P_c cca$	39.625
mZ_2^-	−1	0	0	$P_c mma$	33.225
mZ_3^-	1	0	0	$P_a bam$	106.772
mZ_4^-	0	1	−1	$P_b bcm$	37.791
$mZ_2^- + mZ_4^-$	−1	1	−1	$P_a 2_1/m$	29.303

S2 Rietveld refinements and fit parameters

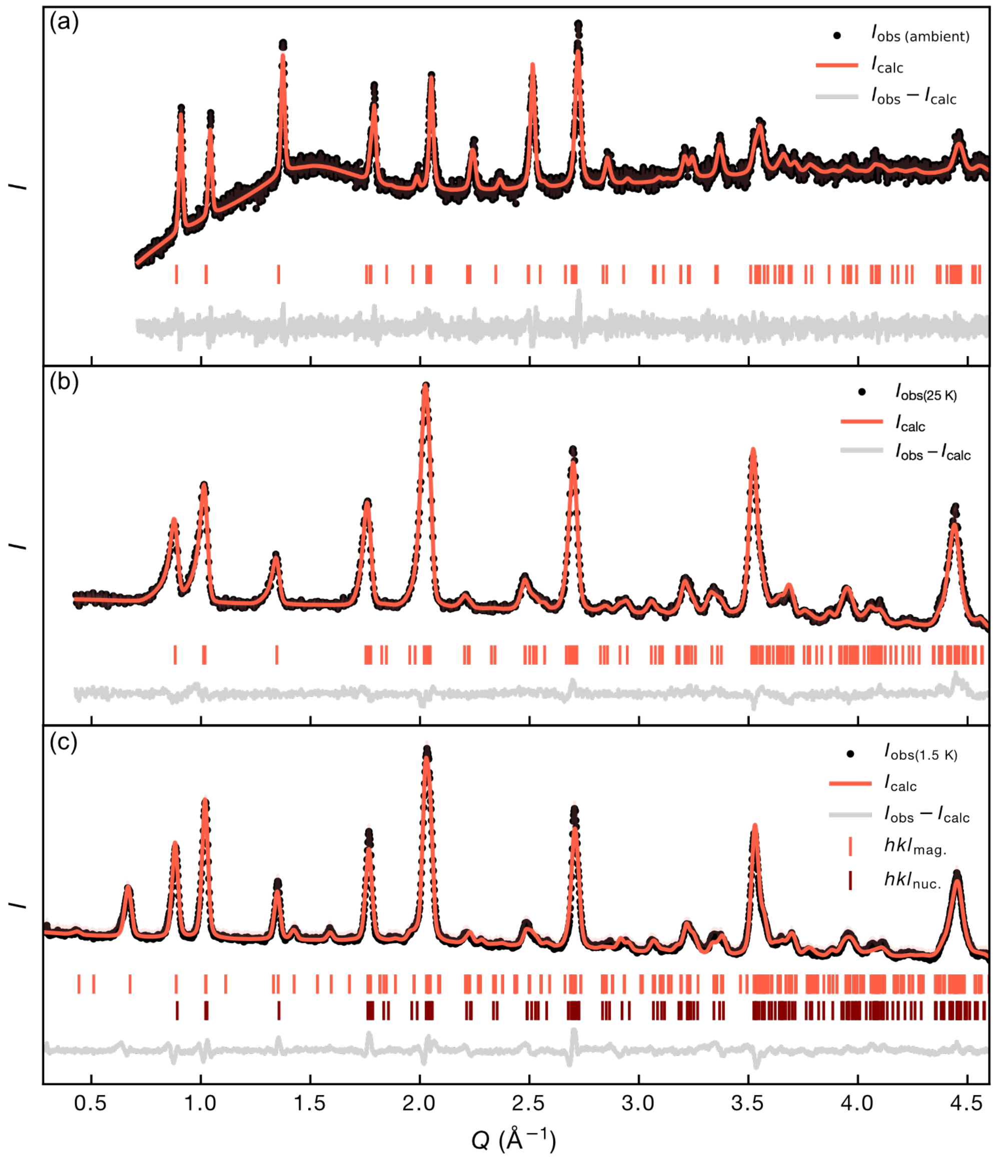

Figure S2: Rietveld refinement[6] of (a) powder X-ray Cu Kα_1 radiation data ($\lambda = 1.5406$ Å) at ambient temperature and powder neutron diffraction data collected at (b) 25 K, (c) 1.5 K* and $\lambda = 2.41$ Å of $FeCl_2$(pym). The unit cell parameters and isotropic displacement parameters were allowed to refine freely, however the atomic coordinates were fixed to those determined from single-crystal XRD. *Simultaneous Rietveld refinement of the nuclear structure and magnetic ground state, that is, the magnetic moment on the Fe was also allowed to refine.

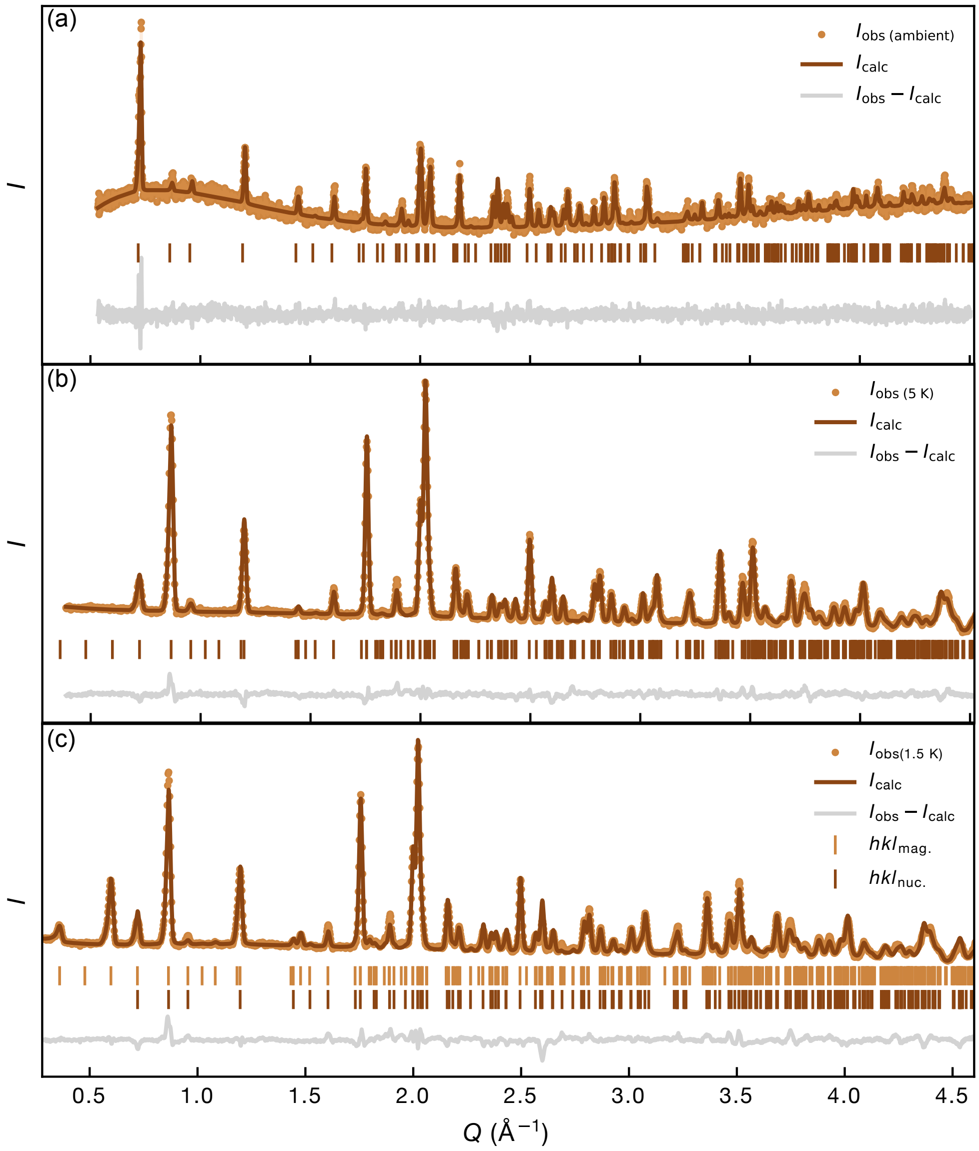

Figure S3: Rietveld refinement[6] of (a) powder X-ray Cu Kα_1 radiation data (λ = 1.5406 Å) at ambient temperature and powder neutron diffraction data collected at (b) 5 K, (c) 1.5 K* and λ = 2.41 Å of $FeCl_2$(btd−d_4). The unit cell parameters and isotropic displacement parameters were allowed to refine freely, the btd molecule was refined as a rigid body and the Fe and Cl atomic coordinates were fixed to those determined from DFT calculations. *Simultaneous Rietveld refinement of the nuclear structure and magnetic ground state, that is, the magnetic moment on the Fe was also allowed to refine.

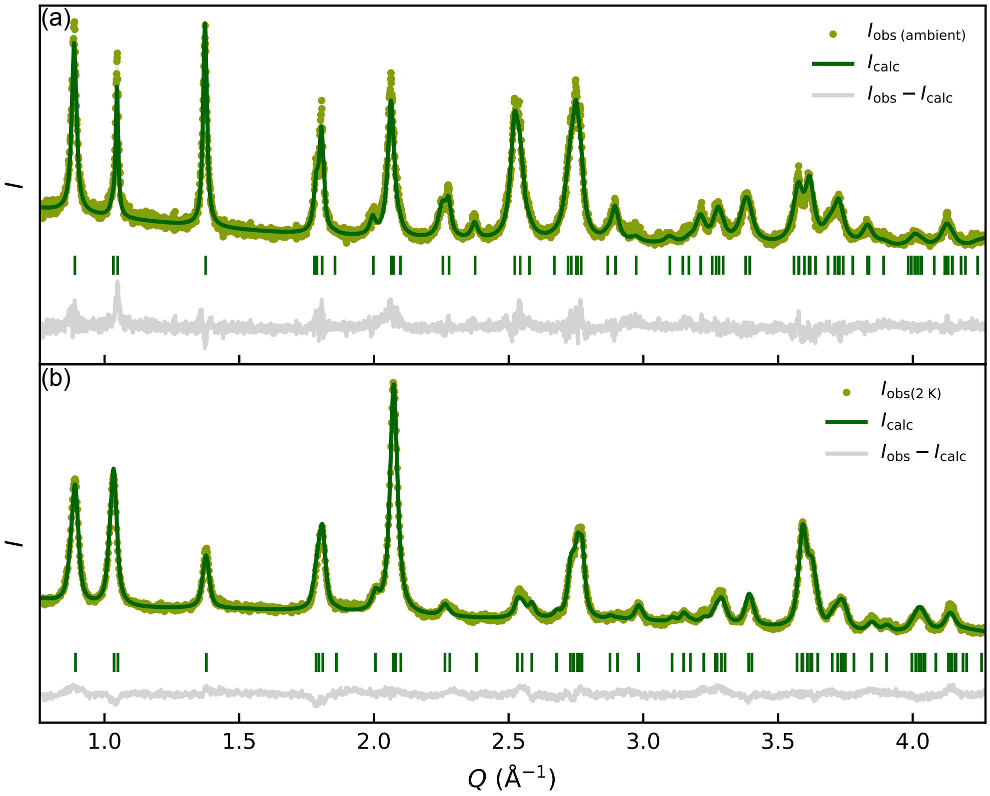

Figure S4: Rietveld refinement[6] of (a) powder X-ray Cu Kα_1 radiation data ($\lambda = 1.5406$ Å) at ambient temperature and (b) powder neutron diffraction data collected at 2 K and $\lambda = 2.41$ Å of $NiCl_2$(pym). The unit cell parameters and isotropic displacement parameters were allowed to refine freely, however the atomic coordinates were fixed to those determined from single-crystal XRD of $NiCl_2$(pym).

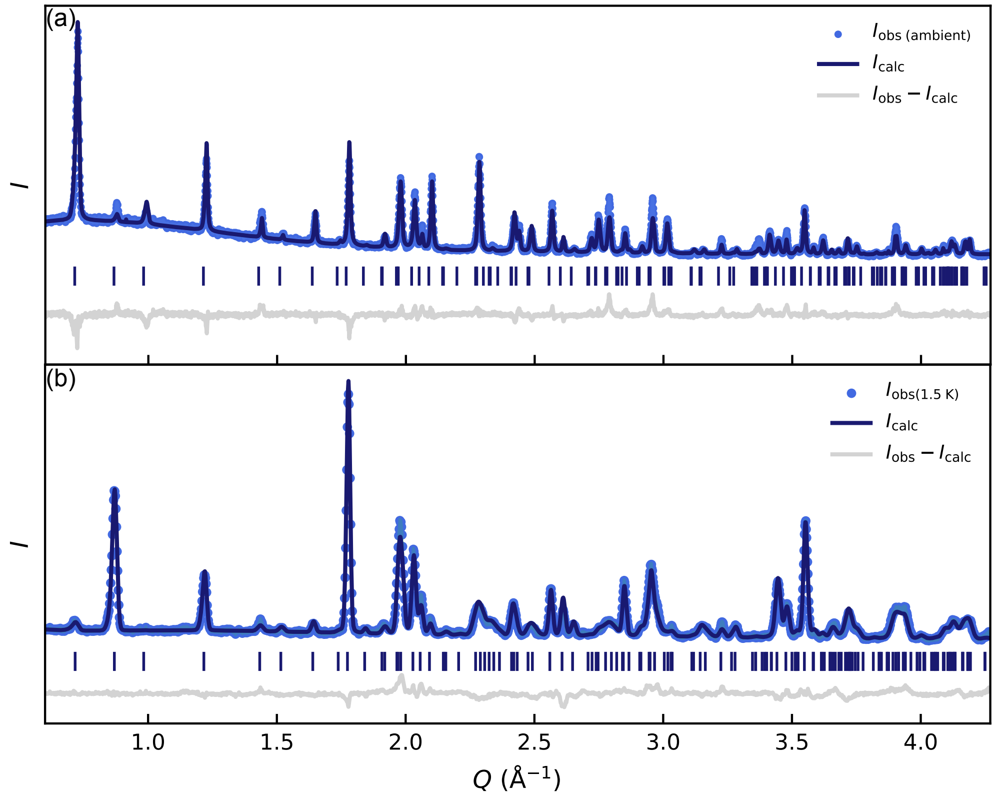

Figure S5: Rietveld refinement[6] of (a) powder X-ray Cu Kα_1 radiation data ($\lambda = 1.5406$ Å) at ambient temperature and (b) powder neutron diffraction data collected at 1.5 K and $\lambda = 2.41$ Å of $NiCl_2$(btd$-d_4$). The unit cell parameters and isotropic displacement parameters were allowed to refine freely, the btd molecule was refined as a rigid body and the Ni and Cl atomic coordinates were fixed to those determined from DFT calculations.

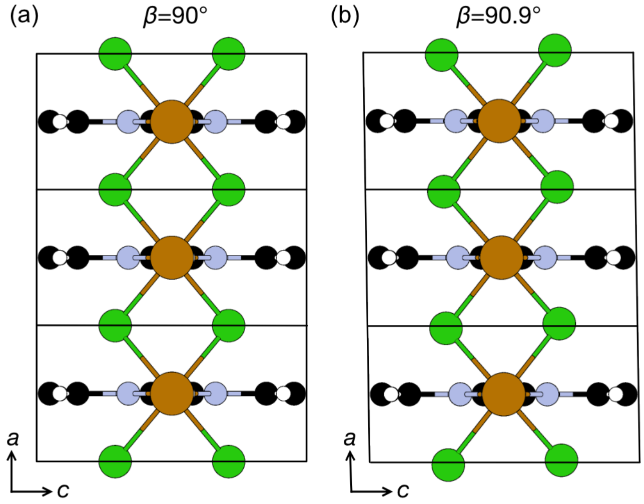

Figure S6: The crytsal structure of $FeCl_2$(pym) viewed along the *b*-axis in the (a) high temperature and (b) low temperature phases.

Table S2: Crystal data and single-crystal XRD structural refinement parameters. Bond lengths for $FeCl_2$(pym).

	$FeCl_2$(pym)
MW (g mol^{-1})	206.84
Crystal system	Orthorhombic
Space group	$P\,m\,m\,b$
a (Å)	3.5644(2)
b (Å)	12.2995(4)
c (Å)	7.0800(3)
V (Å^3)	310.39(2)
T (K)	120.01(10)
Z	2
R_{int}	5.52
R_1	2.51
wR_2	7.08
GOF	1.124
$\Delta\rho_{\text{max}}$,$\Delta\rho_{\text{min}}$/e Å^{-3}	0.4, -0.3

Bond length	r (Å)
Fe-Cl	2.4866(5)
Fe-N	2.205(2)
Fe-N	2.206(2)
N-C1	1.335(3)
N-C2	1.342(4)
C2-C3	1.375(4)

Table S3: Refined lattice parameters from powder XRD.

	$FeCl_2$(pym)	$FeCl_2$(btd$-d_4$)	$NiCl_2$(pym)	$NiCl_2$(btd$-d_4$)
MW (g mol^{-1})	171.39	266.92	174.23	269.77
Crystal system	Ortho-rhombic	Monoclinic	Ortho-rhombic	Monoclinic
Space group	$Pmmb$	$P2_1/m$	$Pmmb$	$P2_1/m$
a (Å)	3.580(6)	3.6190(2)	3.5145(5)	3.5537(1)
b (Å)	12.301(2)	13.1980(8)	11.983(2)	12.8028(4)
c (Å)	7.079(1)	8.7879(7)	7.062(1)	8.7981(4)
β (°)	90	94.501(4)	90	90.120(5)
V (Å^3)	311.84(8)	418.45(5)	297.40(7)	400.29(3)
T (K)	295	295	295	295
Z	2	2	2	2
R_{wp}	2.856	4.539	10.653	7.848
GOF	0.562	1.382	0.984	1.624
R_{Bragg}	0.991	3.162	3.362	4.389
λ (Å)	1.5406	1.5406	1.5406	1.5406
Bond lengths, r (Å)				
M-Cl1	2.492(5)	2.463(8)	2.458(2)	2.441(6)
M-Cl2	2.492(5)	2.554(8)	2.458(2)	2.528(6)
M-N	2.207(16)	2.198(5)	2.197(8)	2.097(7)

Table S4: Refined lattice parameters from PND analysis of the nuclear diffraction. *Magnetic Bragg intensity was accounted for by additional peaks.

	$FeCl_2$(pym)	$FeCl_2$(btd−d_4)	$NiCl_2$(pym)	$NiCl_2$(btd−d_4)
MW (g mol^{-1})	171.39	266.92	174.23	269.77
Crystal system	Ortho-rhombic	Monoclinic	Ortho-rhombic	Monoclinic
Space group	$P2_1/m$	$P2_1/m$	$Pmmb$	$P2_1/m$
a (Å)	3.5656(2)	3.5995(1)	3.4991(3)	3.5417(2)
b (Å)	12.2953(9)	13.1534(4)	11.9710(14)	12.7836(7)
c (Å)	7.0414(8)	8.7465(4)	7.0381(10)	8.7665(7)
β (°)	90.886(8)	95.768(3)	90	90.474(12)
V (Å^3)	308.67(5)	412.02(3)	294.81(6)	396.90(5)
T (K)	12.5	5	2*	2*
Z	2	2	2	2
R_{wp}	1.897	2.783	1.333	3.468
GOF	1.659	2.218	1.646	2.405
R_{Bragg}	1.063	1.860	0.726	2.405
λ (Å)	2.41	2.41	2.41	2.41
Bond lengths, r (Å)				
M-Cl1	2.447(5)	2.413(4)	2.403(4)	2.388(6)
M-Cl2	2.450(5)	2.515(5)	2.403(4)	2.422(6)
M-N	2.220(4)	2.185(1)	2.159(6)	2.060(2)

Table S5: Refined lattice parameters from simultaneous PND analysis of the nuclear and magnetic diffraction.

	$FeCl_2$(pym)	$FeCl_2$(btd−d_4)
MW (g mol^{-1})	171.39	266.92
Crystal system	Monoclinic	Monoclinic
Magnetic Space group	P_a2_1/m	P_a2_1/m
a (Å)	3.5640(3)	3.5995(1)
b (Å)	12.2868(11)	13.1540(5)
c (Å)	14.0859(17)	17.4919(10)
β (°)	90.9399(80)	95.7616(44)
V (Å^3)	616.75(11)	824.037(63)
T (K)	1.5	1.5
Z	4	4
R_{wp}	2.389	3.534
GOF	2.089	2.780
λ (Å)	2.41	2.41
Bond lengths, r (Å)		
M-Cl1	2.434(6)	2.416(5)
M-Cl2	2.475(6)	2.521(6)
M-N	2.239(2)	2.153(2)

S3 Magnetic susceptibility

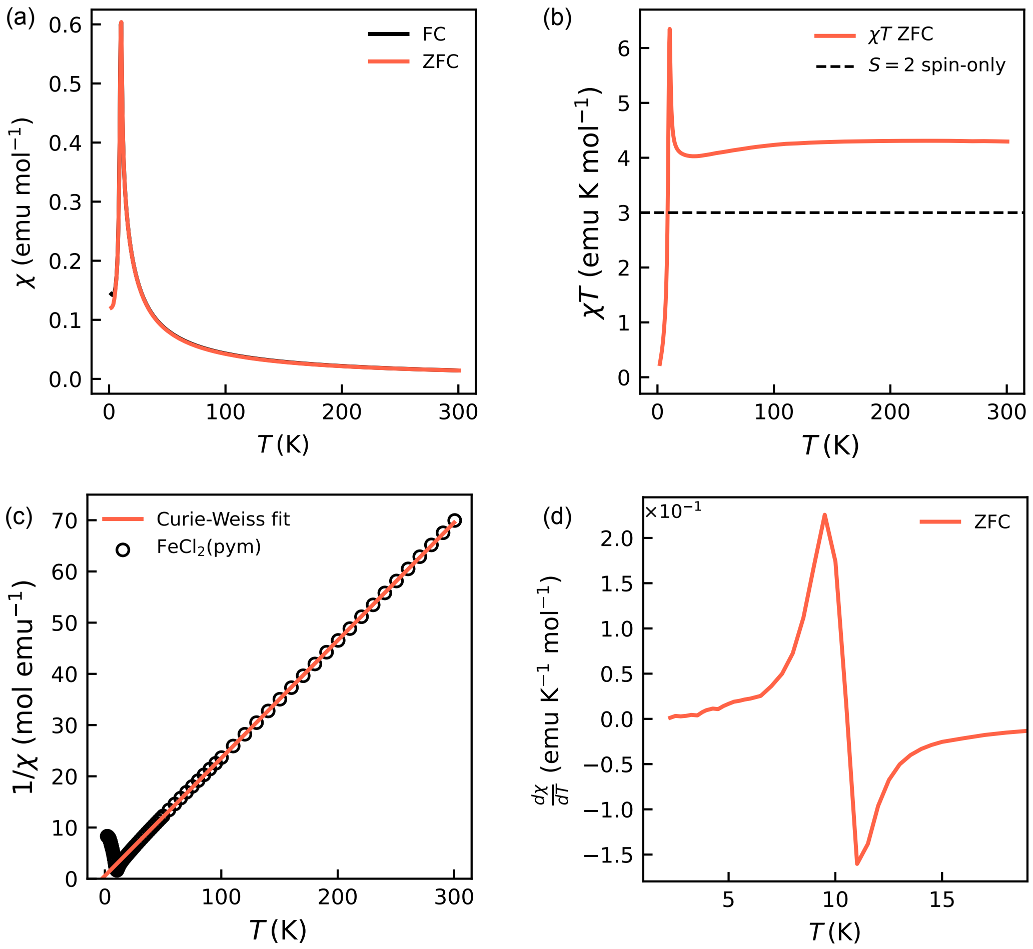

Figure S7: Magnetic susceptibility, χ, measurements of $FeCl_2$(pym). (a) $\chi(T)$ measured in zero-field cooled (ZFC) and field cooled (FC) conditions from 2–300 K. (b) $\chi T(T)$ in ZFC conditions 2–300 K. Dashed line shows the $S = 2$ spin-only limit. (c) $1/\chi(T)$ with Curie-Weiss fit carried out over $300 > T > 100$ K. (d) ZFC $\frac{d\chi}{dT}(T)$ over 2–20 K.

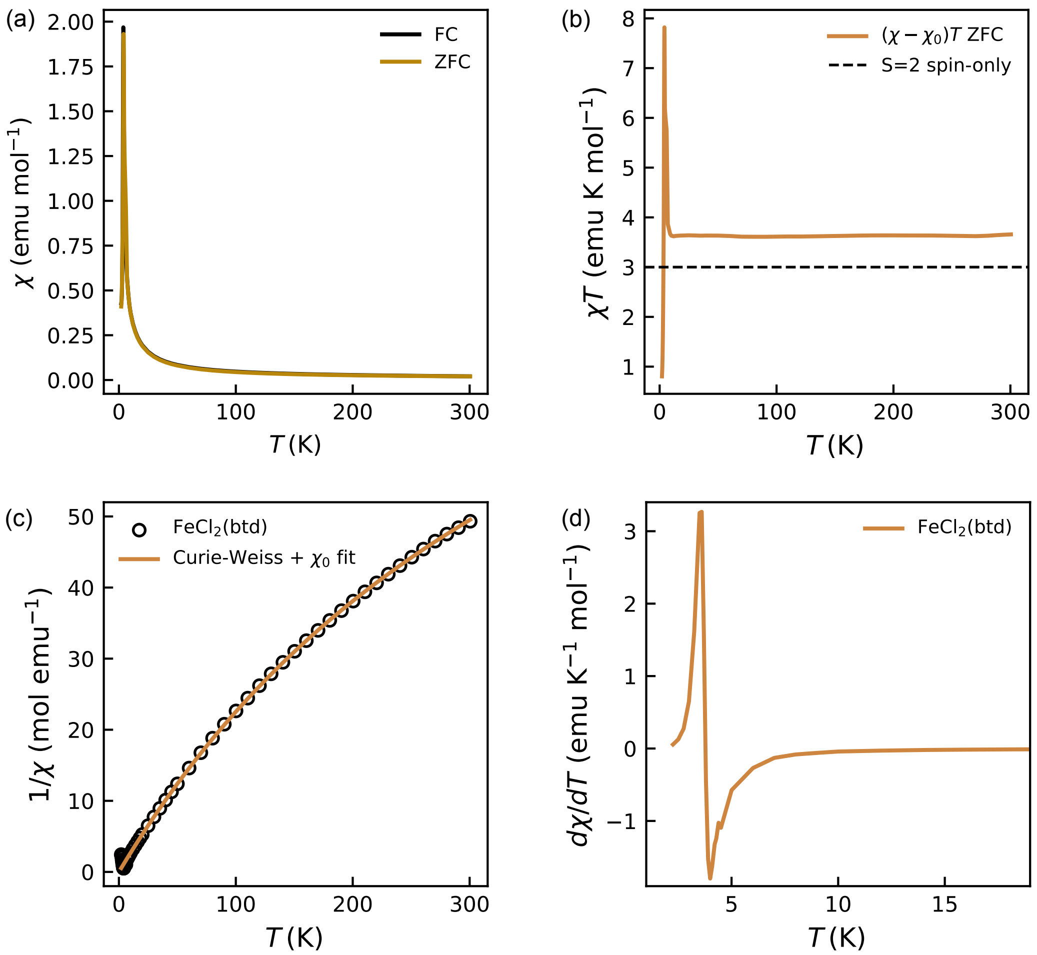

Figure S8: Magnetic susceptibility, χ, measurements of $FeCl_2$(btd). (a) $\chi(T)$ measured in ZFC and FC conditions from 2–300 K. (b) $\chi T(T)$ in ZFC conditions 2–300 K. Dashed line shows the $S = 2$ spin-only limit. (c) $1/\chi(T)$ with modified Curie-Weiss fit including a χ_0 constant carried out over $300 > T > 100$ K. (d) ZFC $\frac{d\chi}{dT}(T)$ over 2–20 K.

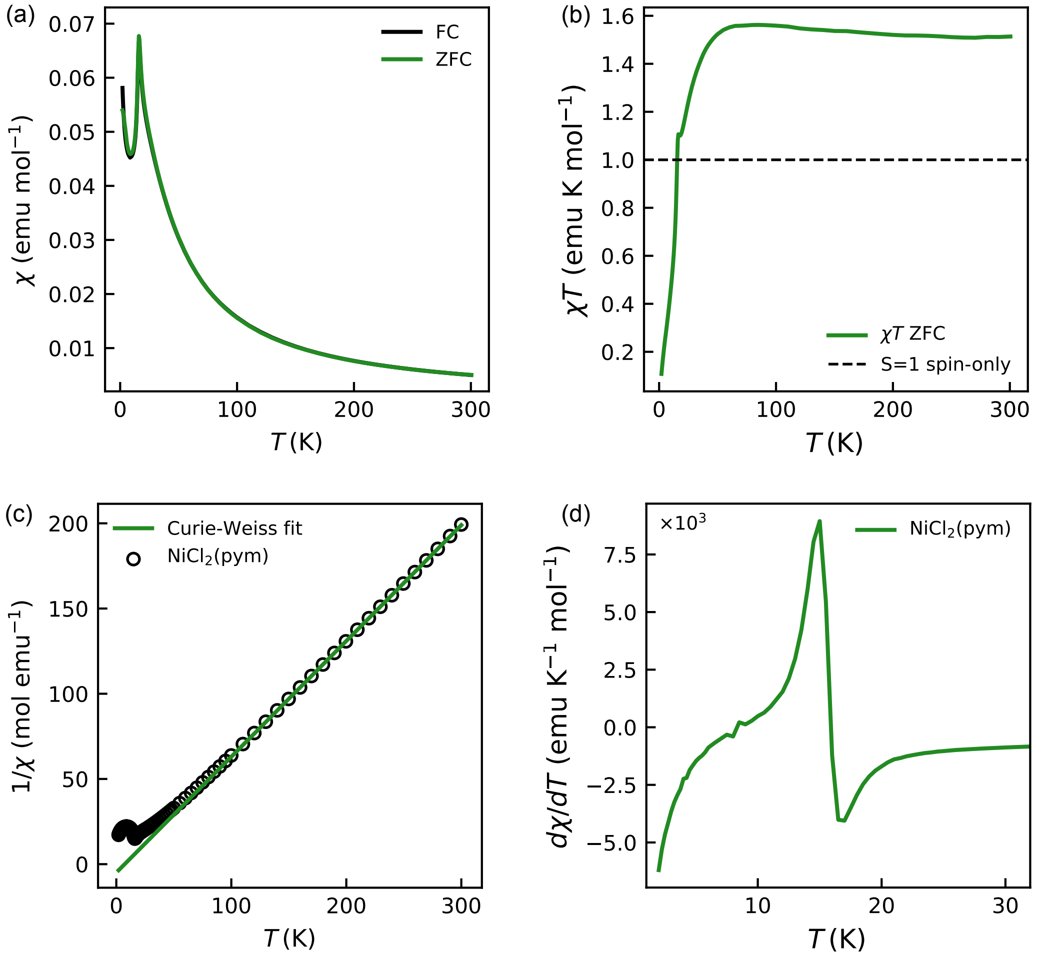

Figure S9: Magnetic susceptibility, χ, measurements of $NiCl_2$(pym). (a) $\chi(T)$ measured in zero-field cooled (ZFC) and field cooled (FC) conditions from 2–300 K. (b) $\chi T(T)$ in ZFC conditions 2–300 K. Dashed line shows the $S = 1$ spin-only limit. (c) $1/\chi(T)$ with Curie-Weiss fit carried out over $300 > T > 100$ K. (d) ZFC $\frac{d\chi}{dT}(T)$ over 2–35 K.

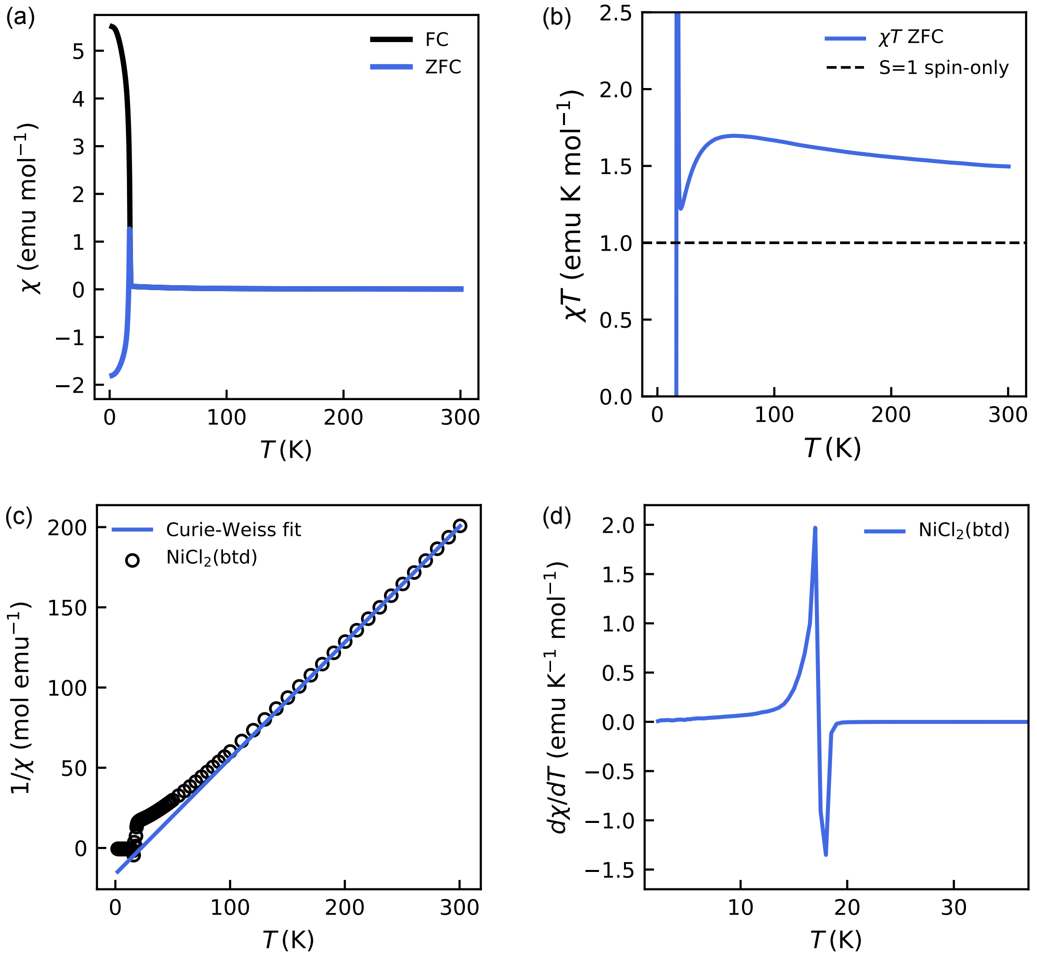

Figure S10: Magnetic susceptibility, χ, measurements of $NiCl_2$(btd). (a) $\chi(T)$ measured in zero-field cooled (ZFC) and field cooled (FC) conditions from 2–300 K. (b) $\chi T(T)$ in ZFC conditions 2–300 K. Dashed line shows the $S = 1$ spin-only limit. (c) $1/\chi(T)$ with Curie-Weiss fit carried out over $300 > T > 100$ K. (d) ZFC $\frac{d\chi}{dT}(T)$ over 2–35 K.

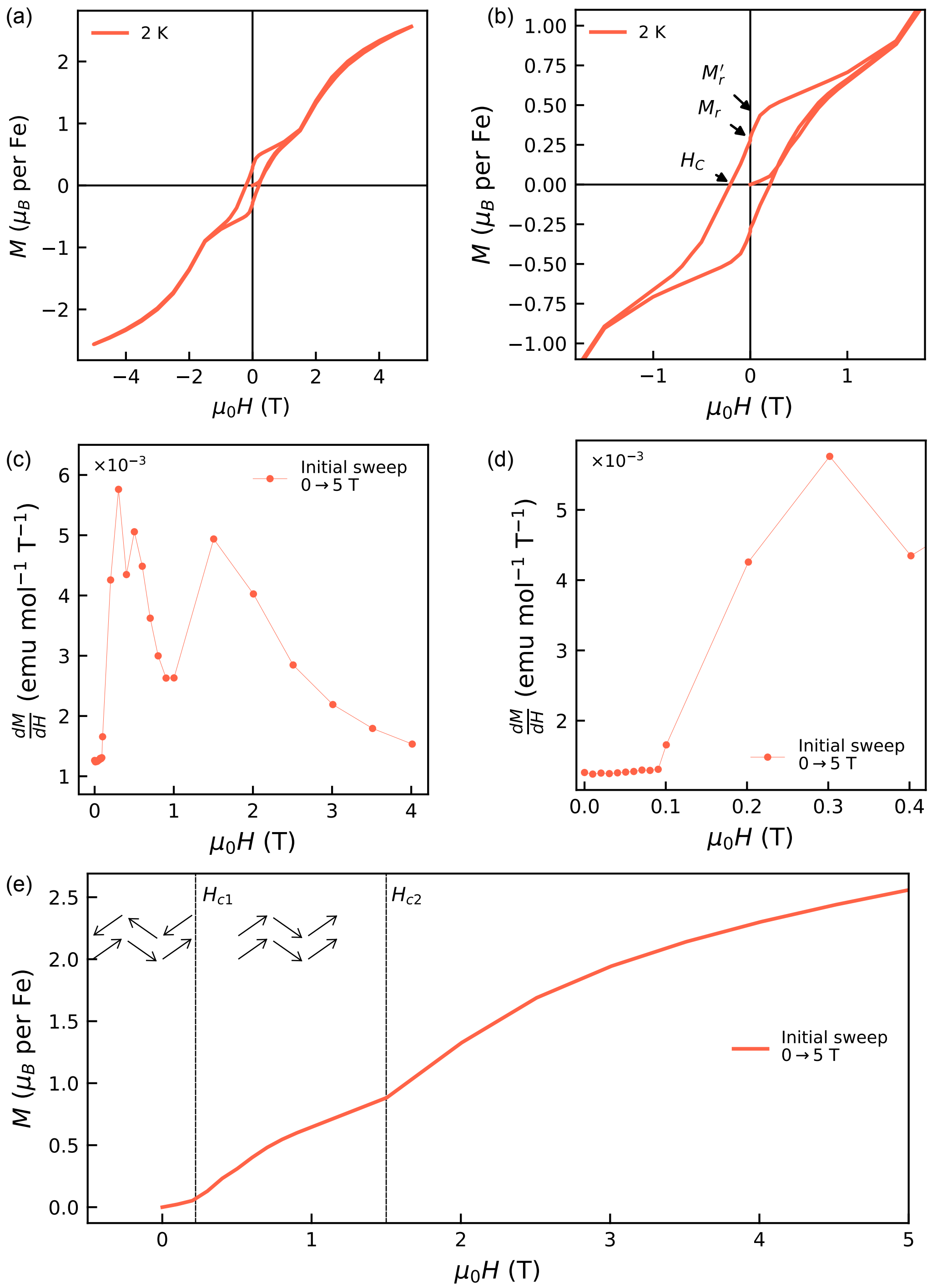

Figure S11: Isothermal magnetisation, $M(H)$ of $FeCl_2$(pym) measured at 2 K between (a) –5 T to 5 T and (b) -1.5 T to 1.5 T. The derivative, $\frac{dM}{dH}(H)$, of the ZFC sweep from (c) 0 T to 5 T and (d) 0 T to 0.1 T. (e) $M(H)$ on the inital sweep from $\mu_0 H = 0$ to 5 T. Insets: A schematic representation of the magnetic phase state viewed along the a-axis.

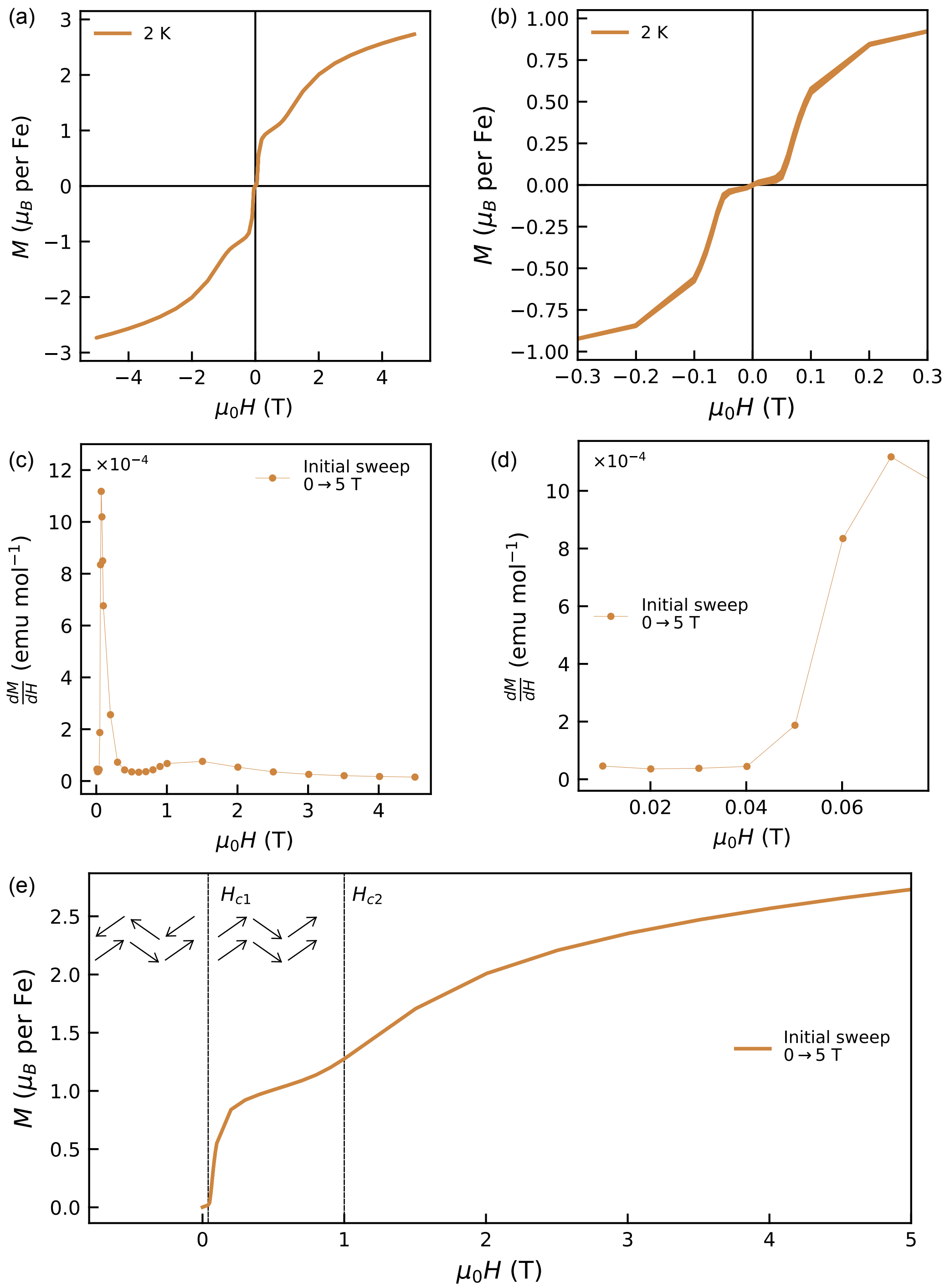

Figure S12: Isothermal magnetisation, $M(\mu_0 H)$ of $FeCl_2(btd{-}d_4)$ measured at 2 K between (a) –5 T to 5 T and (b) -0.3 T to 0.3 T. The derivative, $\frac{dM}{dH}(H)$, of the ZFC sweep from (c) 0 T to 5 T and (d) 0 T to 0.04 T. (e) $M(H)$ on the inital sweep from $\mu_0 H = 0$ to 5 T. Insets: A schematic representation of the magnetic phase state viewed along the a-axis.

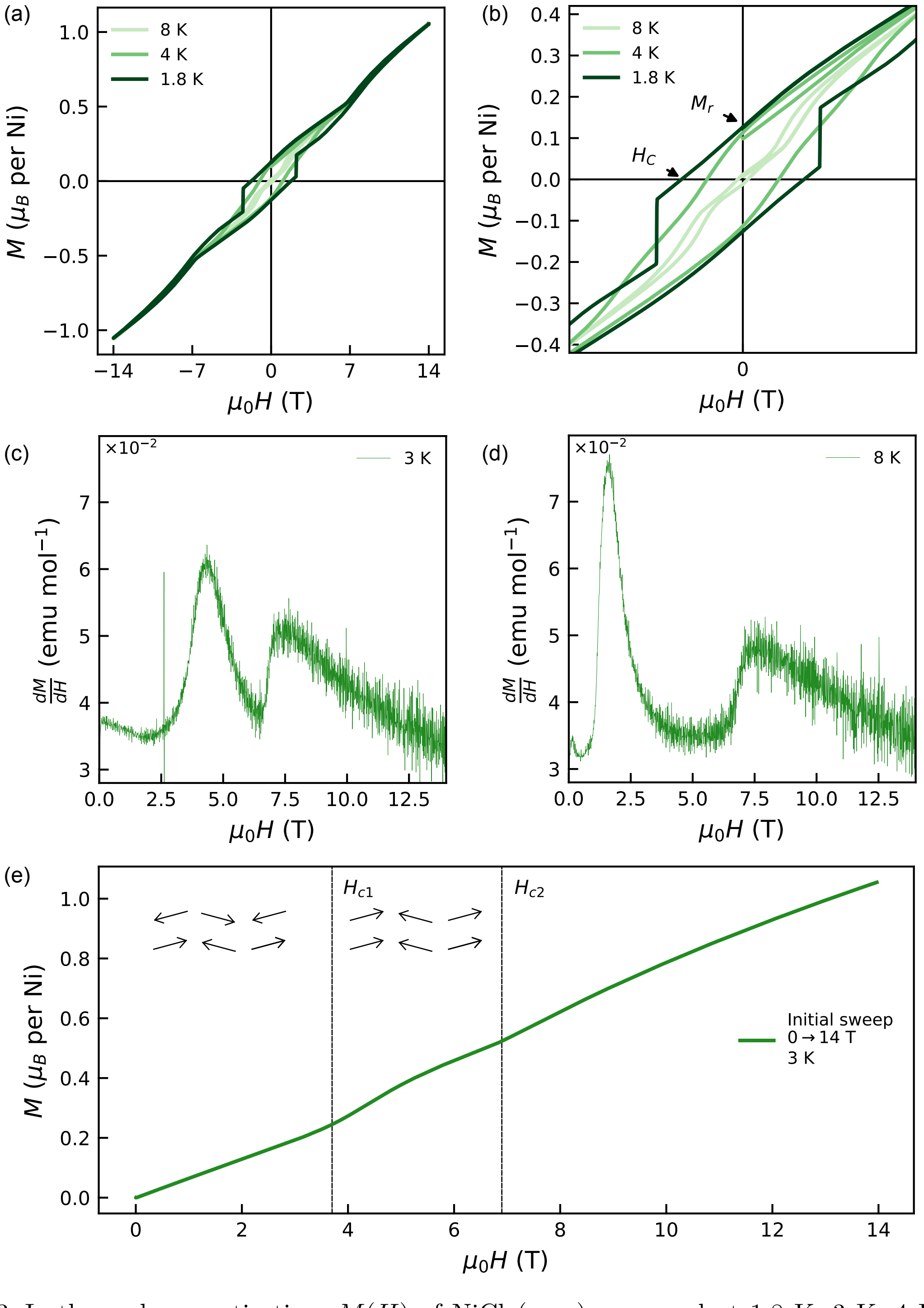

Figure S13: Isothermal magnetisation, $M(H)$ of $NiCl_2$(pym) measured at 1.8 K, 3 K, 4 K and 8 K between (a) –14 T to 14 T and (b) -5 T to 5 T. The derivative, $\frac{dM}{dH}(H)$, of the ZFC sweep from 0 T to 14 T at (c) $T = 3$ K and (d) $T = 8$ K. (e) $M(H)$ on the inital sweep from $\mu_0 H = 0$ to 14 T. Insets: A schematic representation of the magnetic phase state viewed along the a-axis.

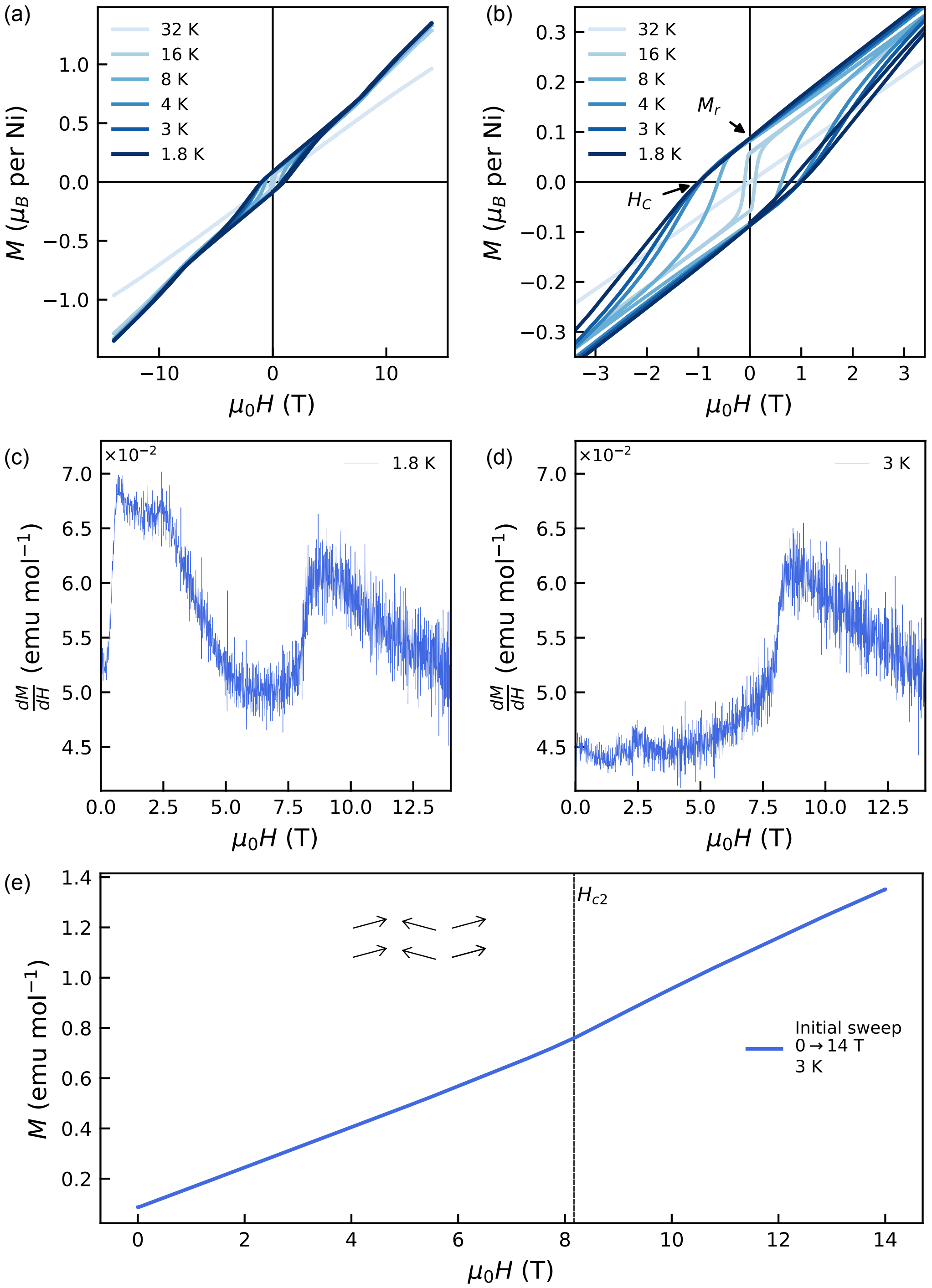

Figure S14: Isothermal magnetisation, $M(H)$ of $NiCl_2$(btd$-d_4$) measured at 1.8 K, 3 K, 4 K, 8 K, 16 K, and 32 K between (a) –14 T to 14 T and (b) -3.4 T to 3.4 T. The derivative, $\frac{dM}{dH}(H)$, of the ZFC sweep from 0 T to 14 T at (c) $T = 1.8$ K where $M(H=0) = -M_r$ and (d) $T = 3$ K where $M(H=0) = M_r$. (e) $M(H)$ on the inital sweep from $\mu_0 H = 0$ to 14 T. Insets: A schematic representation of the magnetic phase state viewed along the a-axis.

S4 Powder neutron diffraction

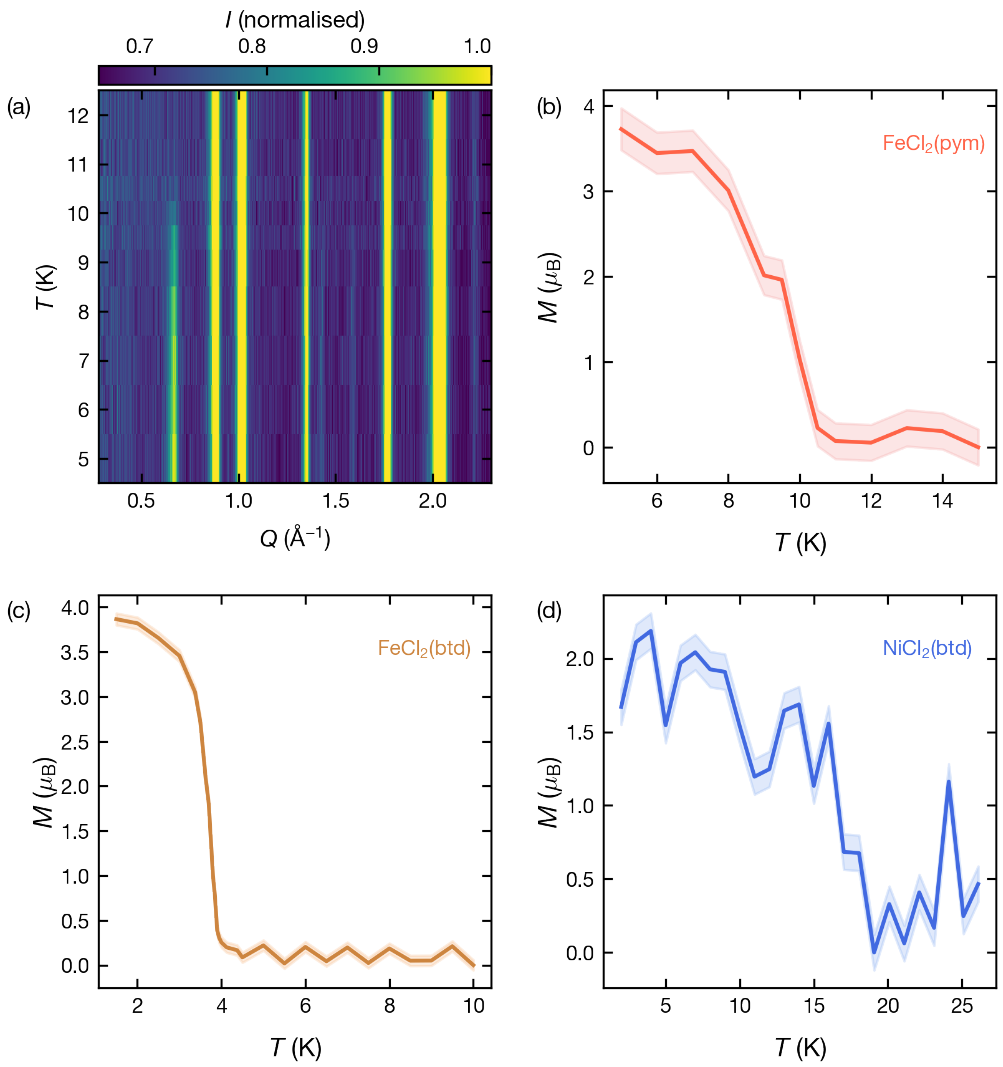

Figure S15: The temperature dependent PND of $FeCl_2$(pym) (a) at $0.27 < Q < 2.3$ $Å^{-1}$ and (b) summed over $0.66 < Q < 0.67$ $Å^{-1}$ at temperatures $5 \leq T \leq 12$ K. (c) The temperature dependent PND of $FeCl_2$(btd) at $Q = 0.60$ $Å^{-1}$ and $1.5 < T < 10$ K. (d) The temperature dependent PND of $NiCl_2$(btd) at $Q = 0.87$ $Å^{-1}$ and $2 < T < 26$ K.

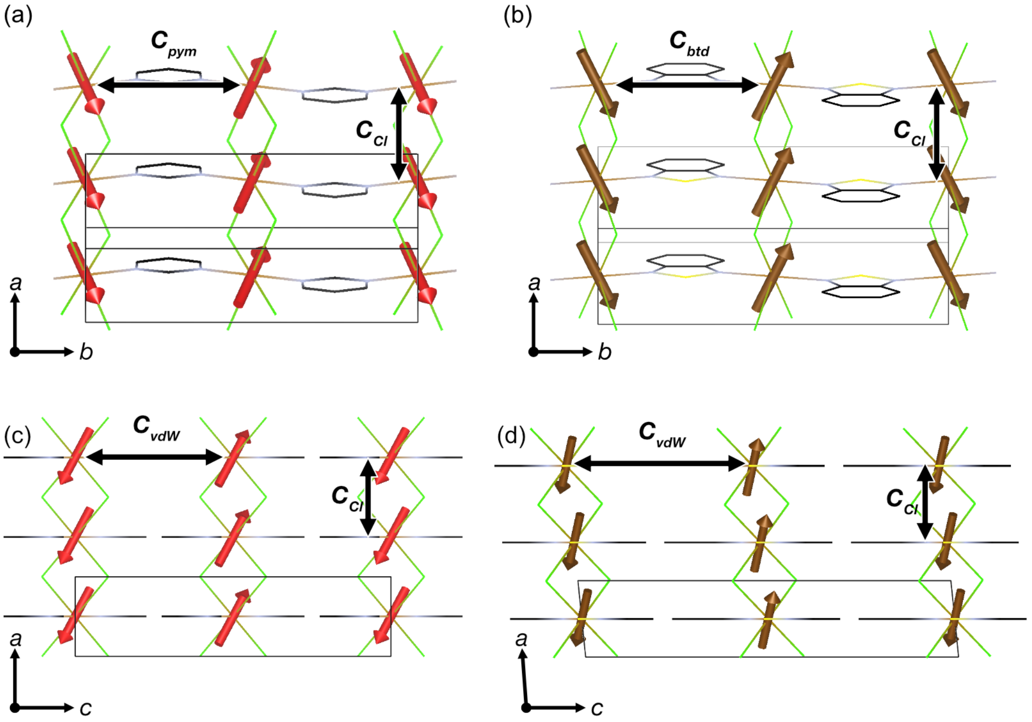

Figure S16: Magnetic ground state of $FeCl_2$(pym) viewed along the (a) c-axis and (c) a-axis, and $FeCl_2$(btd$-$d$_4$) viewed along the (b) c-axis and (d) a-axis. For simplicity the basis vectors are given in relation to the parent nuclear cell. The axes shown correspond to the basis vectors of the parent-like cell used to describe the ordered moment, $M_{a,b,c}$. The magnetic correlations, $\mathcal{C}_X$ (X = Cl, pym, btd and vdW), are described by referring to the chemical connectivity in their respective crystallographic axes.

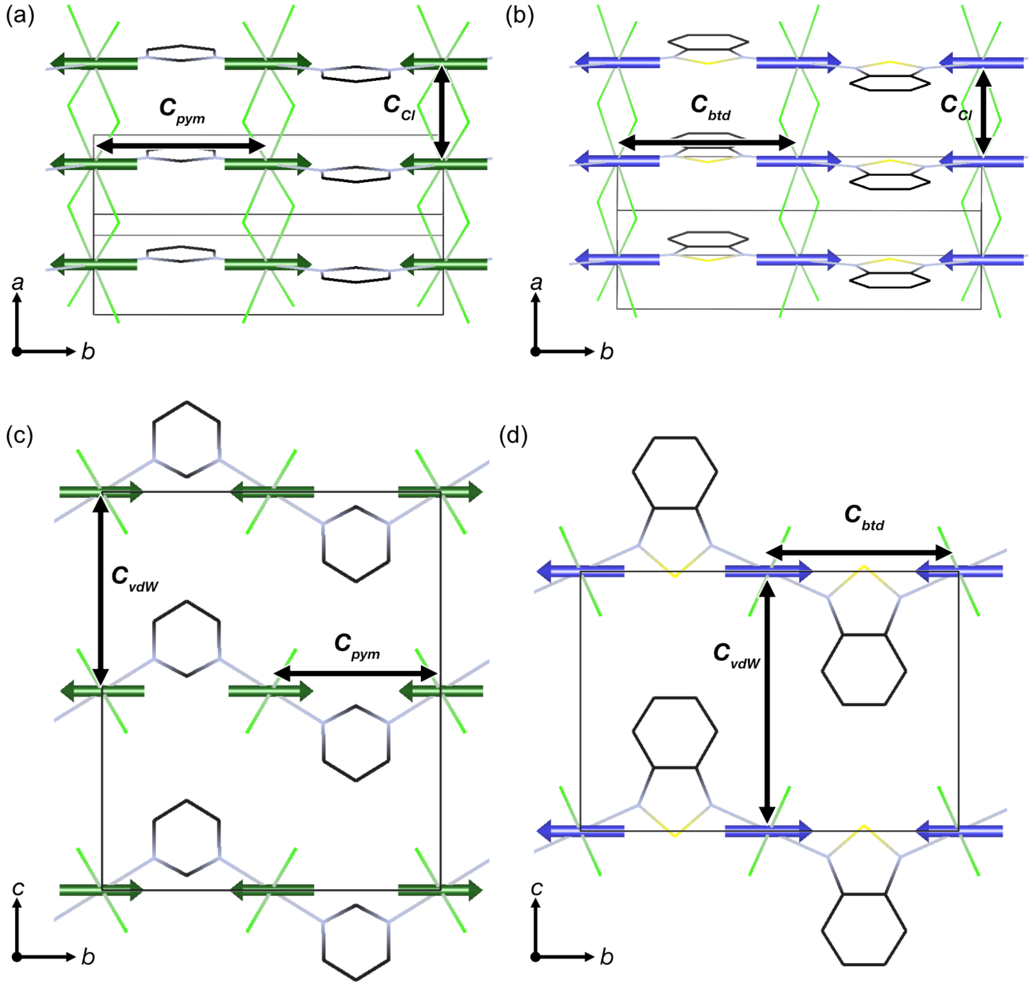

Figure S17: Magnetic ground state of $NiCl_2$(pym) viewed along the (a) c-axis and (c) a-axis, and $NiCl_2$(btd$-$d$_4$) viewed along the (b) c-axis and (d) a-axis. The axes shown correspond to the basis vectors of the parent-like cell used to describe the ordered moment, $M_{a,b,c}$. The magnetic correlations, $\mathcal{C}_X$ (X = Cl, pym, btd and vdW), are described by referring to the chemical connectivity in their respective crystallographic axes.

S5 UV-Vis spectroscopy

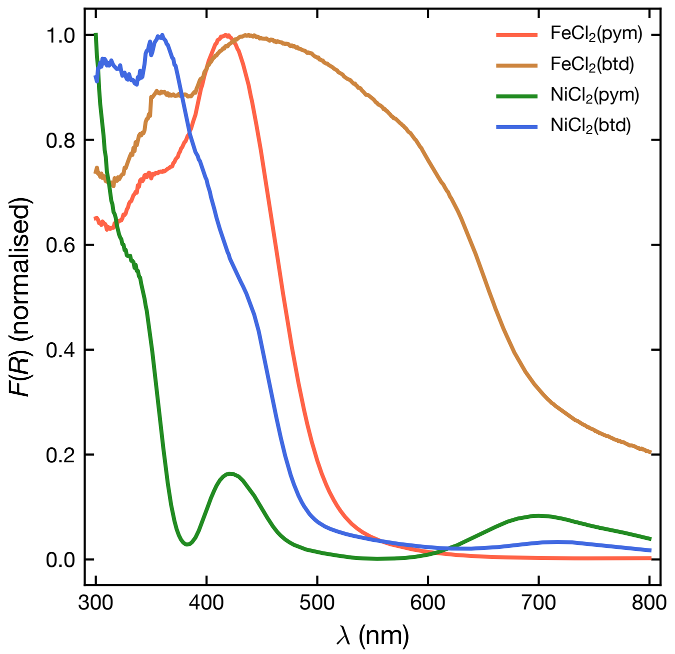

Figure S18: Normalised diffuse reflectance UV-Visible spectra of $FeCl_2$(pym), $FeCl_2$(btd), $NiCl_2$(pym) and $NiCl_2$(btd). $F(R)$ is the Kubelka—Munk transformation of the raw diffuse reflectance spectrum. $F(R)$ for each material was normalised with the strongest absorbance at $300 < \lambda < 800$ nm set to $F(R) = 1$.

S6 Density functional theory

S6.1 DFT geometry optimizations

The experimental structures were refined using collinear spin-polarized plane-wave DFT+U geometry optimizations. The PBE+U+MBD* refined structures for collinear calculations are reported in Tab. S6.

Table S6: Calculated lattice parameters from collinear PBE+U+MBD* DFT.

	$FeCl_2$(pym)	$FeCl_2$(btd)		$NiCl_2$(pym)	$NiCl_2$(btd)
U (eV)	2	0	2	6	6
a (Å)	3.57	3.54	3.61	3.61	3.58
b (Å)	12.28	12.89	13.00	11.66	12.72
c (Å)	7.05	8.64	8.66	6.97	8.57
α (°)	89.87	90.00	90.00	90.00	90.00
β (°)	90.06	93.38	94.11	90.00	91.36
γ (°)	90.24	90.00	90.00	90.00	90.00
V (Å^3)	309.29	394.14	405.11	293.54	391.10
Bond lengths, r (Å)					
M-Cl1	2.45	2.44	2.44	2.54	2.44
M-Cl2	2.57	2.48	2.55	2.54	2.45
M-N	2.21	2.15	2.19	2.01	2.11

S6.2 DFT calculated superexchange

Table S7: Calculated magnetic superexchange from collinear PBE+U+MBD*.

	$NiCl_2$(pym)			$NiCl_2$(btd)		
U (eV)	3	6	9	3	6	9
J_{Cl} (meV)	5.42(6)	2.55(4)	0.93(2)	4.46(1)	2.52(1)	1.15(1)
J_L (meV)	-3.80(6)	-2.50(4)	-1.28(2)	-1.46(1)	-0.83(1)	-0.42(1)
J_{vdW} (meV)	-0.06(6)	-0.05(4)	-0.03(2)	0.00(1)	0.00(1)	0.01(1)

S6.3 Band-structure and density of states

The electronic band structures along with their respective projected density of states were calculated for the collinear magnetic ground-state configurations of $FeCl_2$(pym), $NiCl_2$(pym), and $NiCl_2$(btd) with PBE+U+MBD* and PBE+MBD* only for $FeCl_2$(btd). Although the DFT+U geometry optimization was successfully carried with $U = 2$ eV for $FeCl_2$(btd), further calculations using U on this state were not possible, as it converged poorly, particularly in the presence of different magnetic states, and so we have shown the $U = 0$ states here.
The ground state band structure shows the presence of a thermal band gap of 1.51,eV for $FeCl_2$(pym), 2.47,eV for $NiCl_2$(pym) and 1.52,eV for $NiCl_2$(btd). The band structure for $FeCl_2$(btd) shows an unphysical metallic nature with a gap below the Fermi energy corresponding to 0.85 eV.

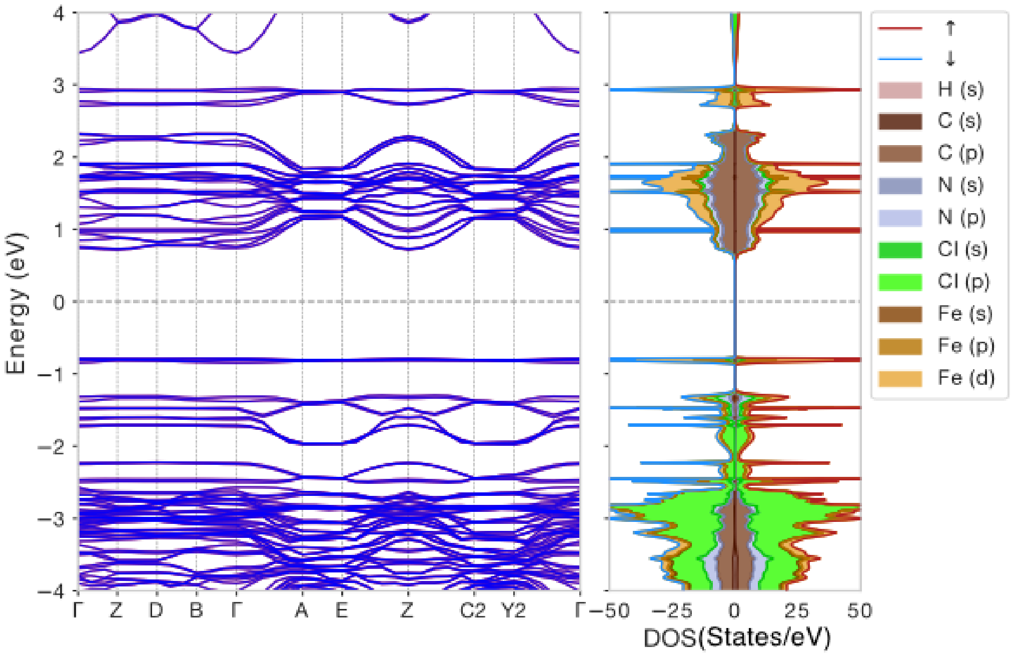

Figure S19: The band structure and projected density of states of $FeCl_2$(pym) using CASTEP 23.1 at the PBE+U+MBD* (U = 2 eV) level. Density of states has been projected over LCAO states using OptaDOS.

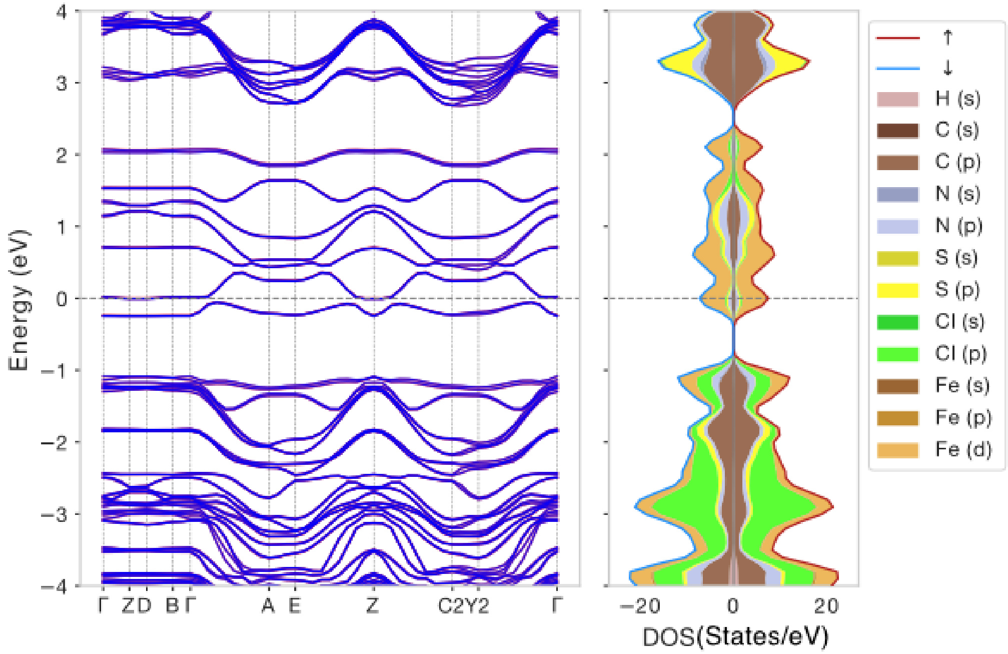

Figure S20: The band structure and projected density of states of $FeCl_2$(btd–d_4) using CASTEP 23.1 at the PBE+MBD* level. Density of states has been projected over LCAO states using OptaDOS.

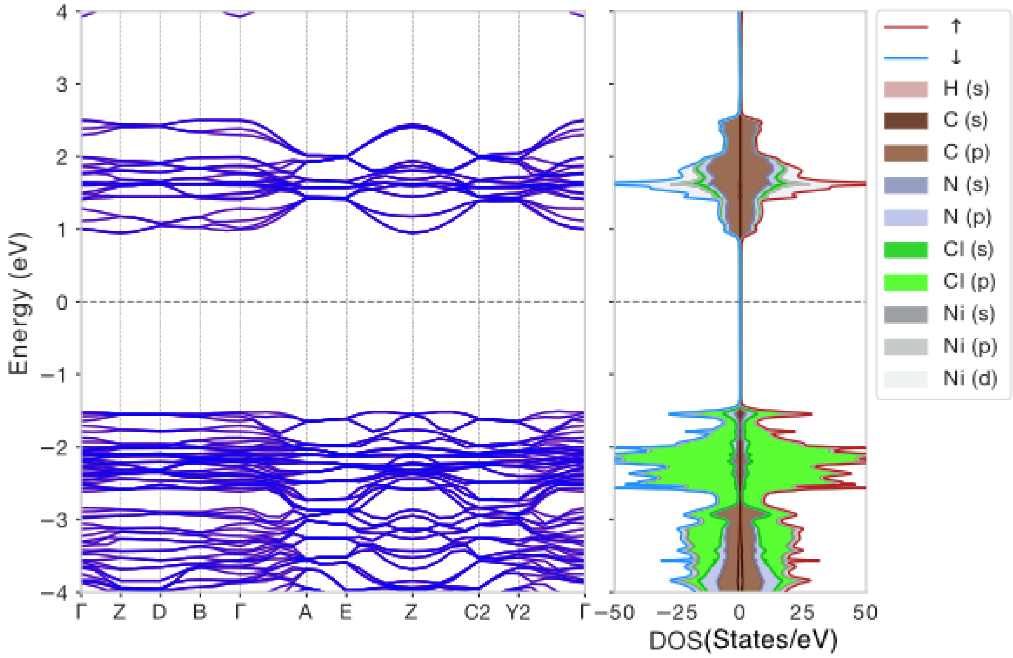

Figure S21: The band structure and projected density of states of $NiCl_2$(pym) using CASTEP 23.1 at the PBE+U+MBD* (U = 6 eV) level. Density of states has been projected over LCAO states using OptaDOS.

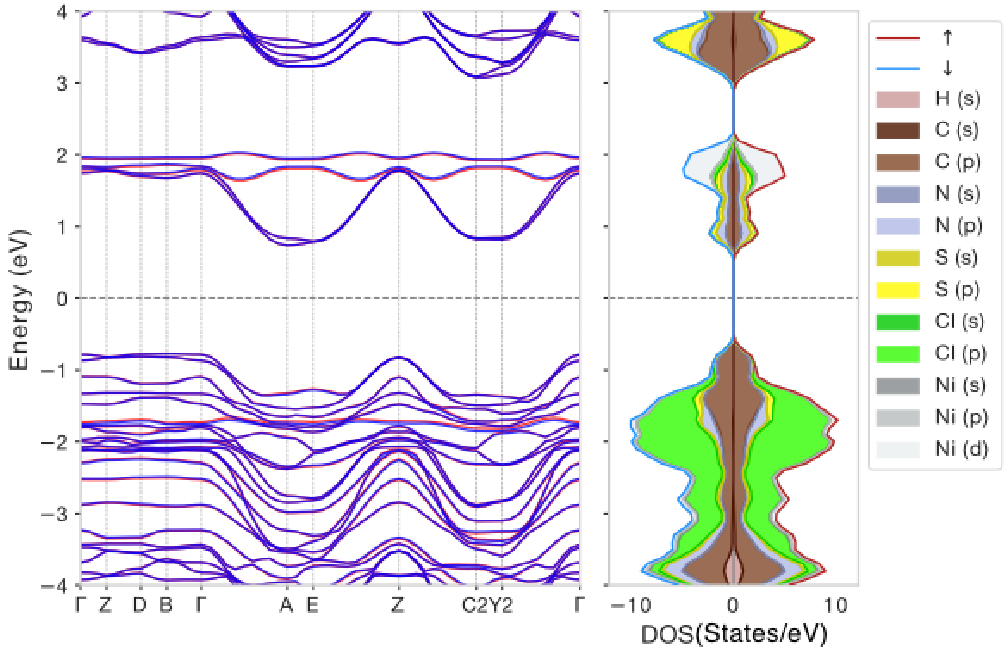

Figure S22: The band structure and projected density of states of $NiCl_2(btd–d_4)$ using CASTEP 23.1 at the PBE+U+MBD* (U = 6 eV) level. Density of states has been projected over LCAO states using OptaDOS.

S6.4 Spin density

The ground-state spin density from collinear PBE+U+MBD* calculations has been visualised and presented in Figures S23, S24, S25 and S26.

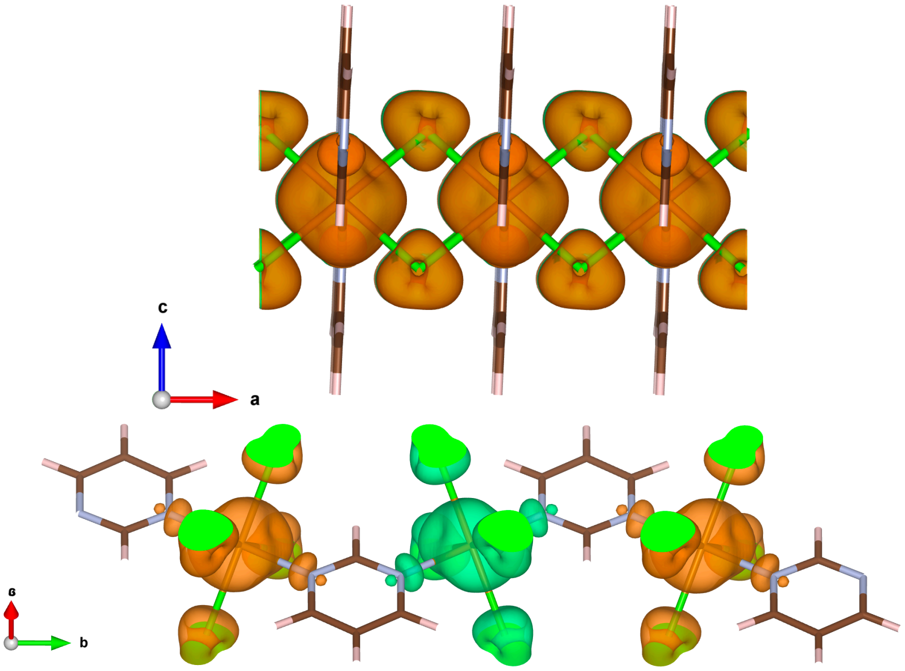

Figure S23: Spin density isosurfaces (0.015 e $Å^{-3}$) of $FeCl_2(pym)$ highlighting the Fe-Cl chain and Fe-pym chain, derived from CASTEP PBE+U+MBD* (U_{eff} = 2 eV) and c2x calculations. The colour scheme differentiates between positive (orange) and negative (green) spin densities.

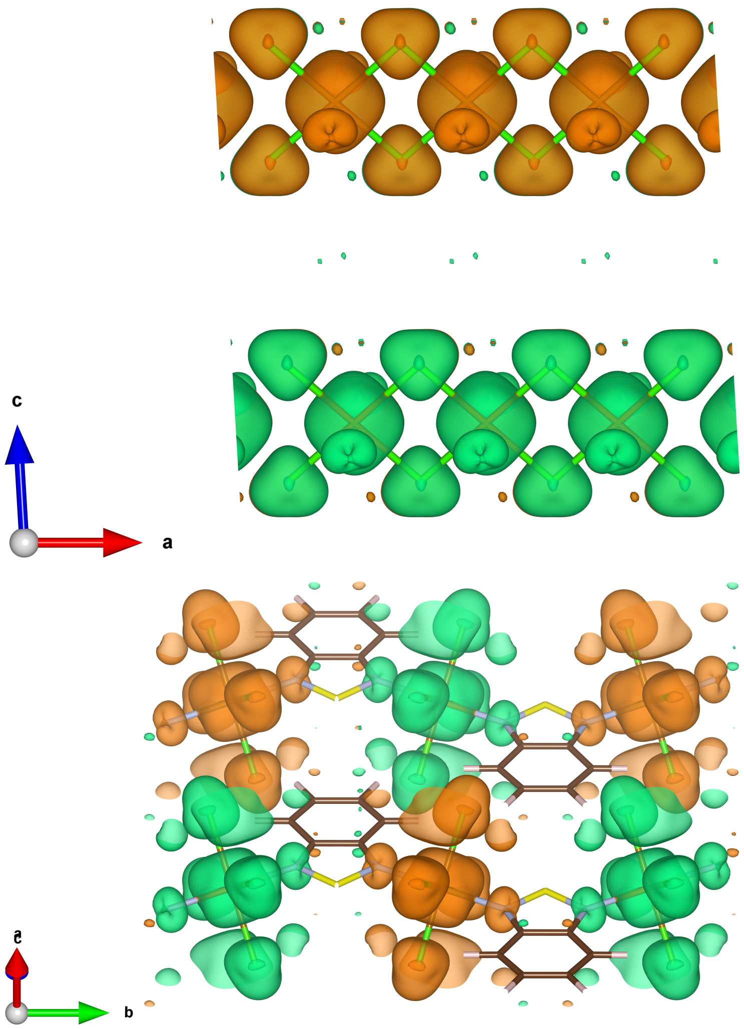

Figure S24: Spin density isosurfaces (0.015 e Å^{-3}) of $FeCl_2$(btd–d_4) highlighting the Fe-Cl chain and Fe-btd chain, derived from CASTEP PBE+MBD* and C2X calculations. The colour scheme differentiates between positive (orange) and negative (green) spin densities.

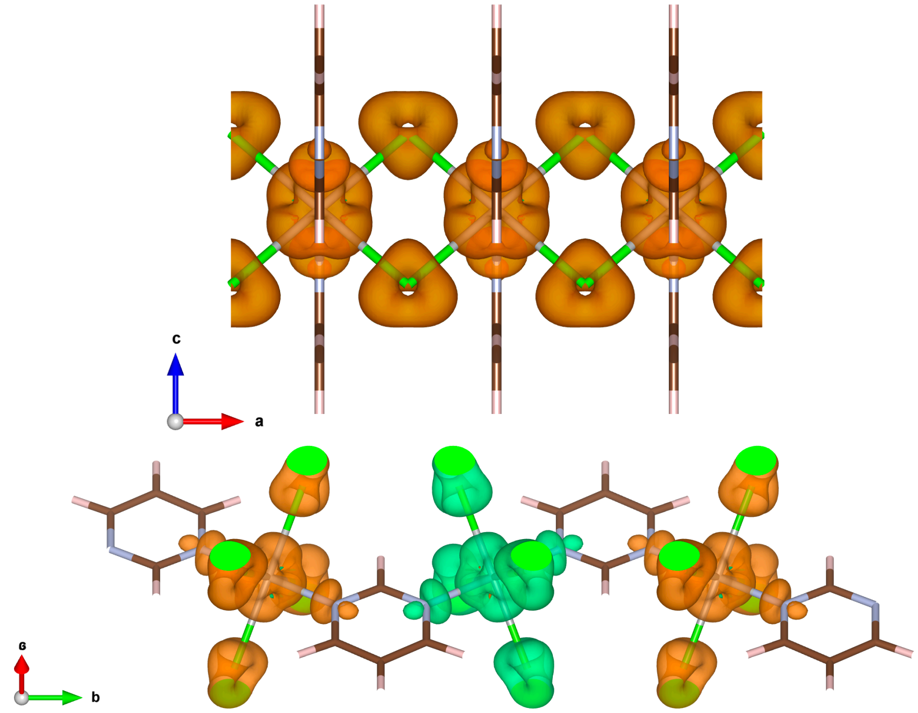

Figure S25: Spin density isosurfaces (0.015 e Å^{-3}) of $NiCl_2$(pym) highlighting the Ni-Cl chain and N-pym chain, derived from CASTEP PBE+U+MBD* ($U_{\mathtt{eff}}$ = 6 eV) and C2X calculations. The colour scheme differentiates between positive (orange) and negative (green) spin densities.

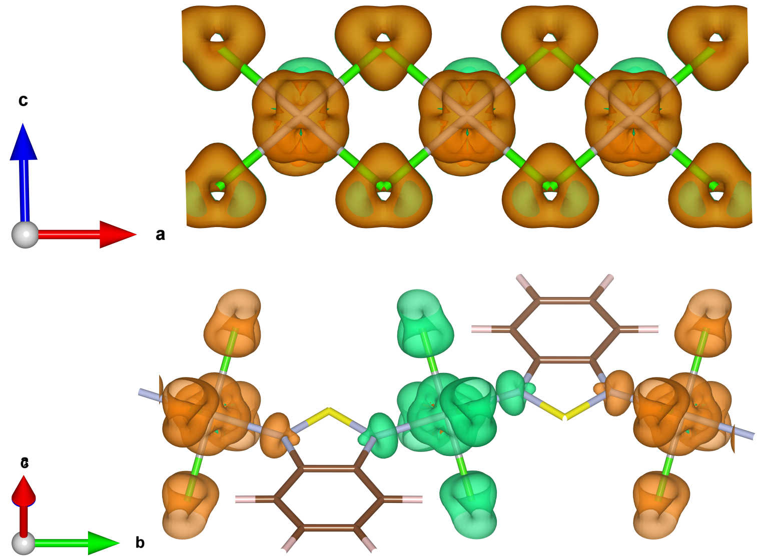

Figure S26: Spin density isosurfaces (0.015 e Å^{-3}) of $NiCl_2$(btd–d_4) highlighting the Ni-Cl chain and N-btd chain, derived from CASTEP PBE+U+MBD* ($U_{\mathtt{eff}}$ = 6 eV) and C2X calculations. The colour scheme differentiates between positive (orange) and negative (green) spin densities.

S6.5 Visualising Kohn-Sham orbitals

Kohn-Sham orbitals from collinear PBE+U+MBD* calculations have been visualised and presented in Figures S27, S29 and S30 and collinear PBE+MBD* calculations in Figure S28.

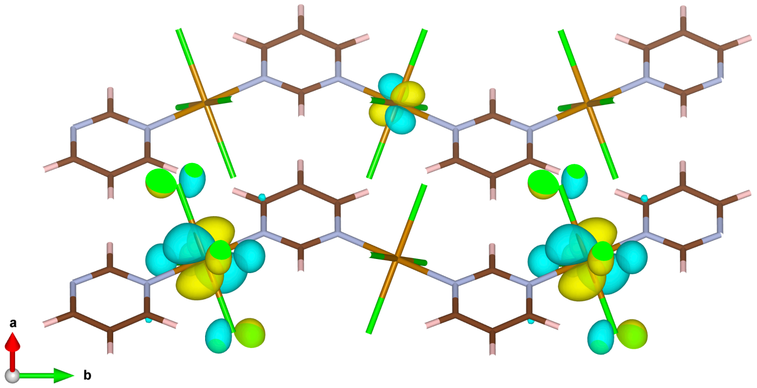

(a) HOMO

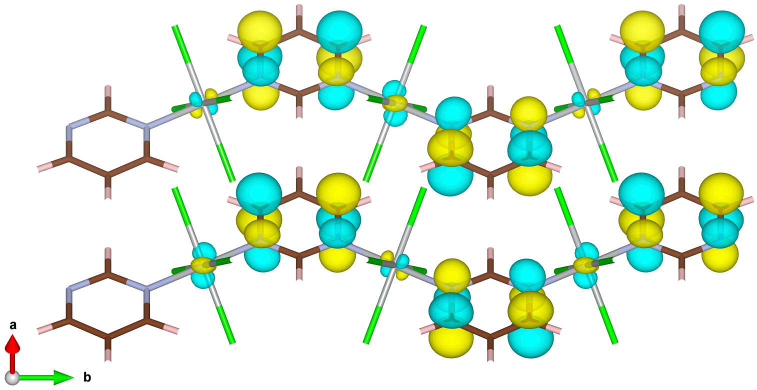

(b) LUMO

Figure S27: Visualisation of HOMO and LUMO of $FeCl_2$(pym) calculated at the Γ point using the DFT package CASTEP along with C2X. The yellow and blue colours correspond to the two spin channels.

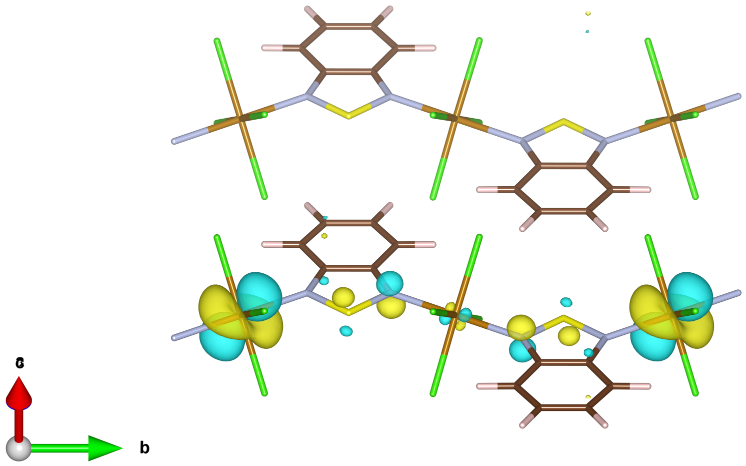

(a) At Fermi energy

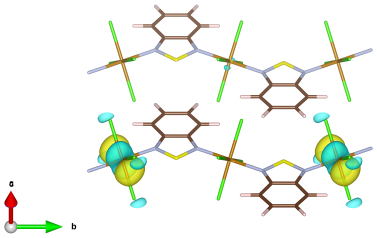

(b) pseudo LUMO

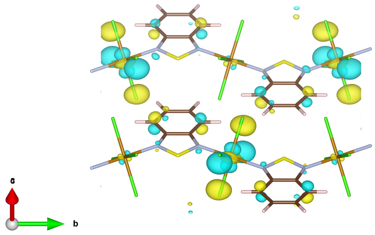

(c) pseudo HOMO

Figure S28: Visualisation of orbitals at the Fermi energy, above the sub-Fermi gap, and below the sub-Fermi gap of $1 \times 1 \times 2$ $FeCl_2$(btd–d_4) supercell calculated at the Γ point using the DFT package CASTEP along with C2X. The yellow and blue colours correspond to the two spin channels.

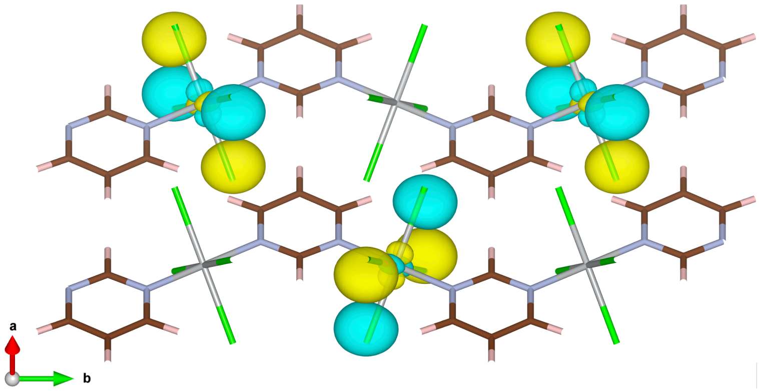

(a) HOMO

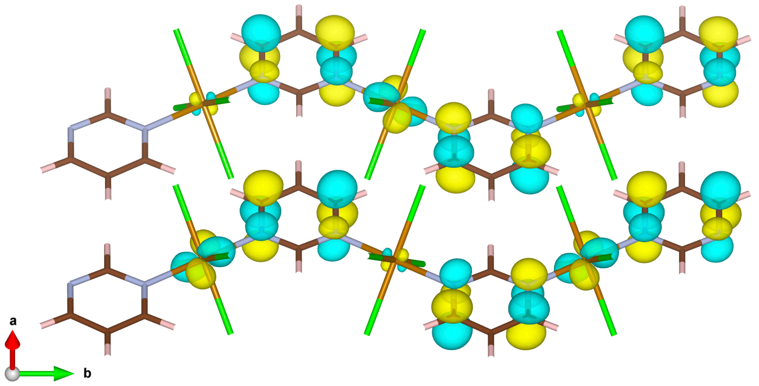

(b) LUMO

Figure S29: Visualisation of HOMO and LUMO of $NiCl_2$(pym) calculated at the Γ point using the DFT package CASTEP along with C2X. The yellow and blue colours correspond to the two spin channels.

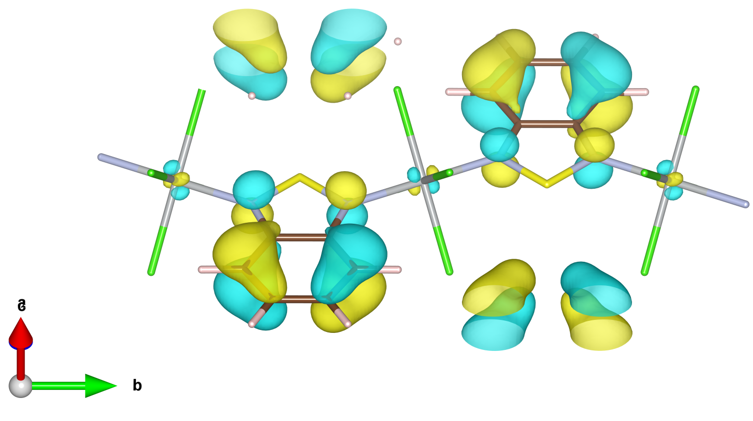

(a) HOMO

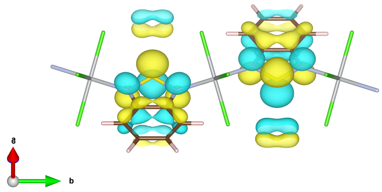

(b) LUMO Γ

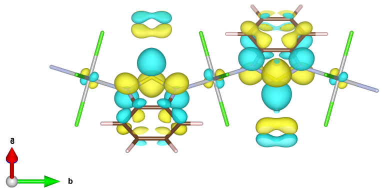

(c) LUMO A

Figure S30: Visualisation of HOMO and LUMO of $NiCl_2$(btd–d_4) unit cell calculated at the Γ point for HOMO and Γ & A point for LUMO using the DFT package CASTEP along with C2X. The yellow and blue colours correspond to the two spin channels.

S6.6 Non-collinear relativistic DFT

The non-collinear magnetism in these vdW MOM is more accurately represented by non-collinear DFT. We tried to find the spin vectors of the magnetic ground-state configurations for our systems. However, we found that optimisation of the spin direction was not possible, as due to the very small energy differences spins did not relax from their initial configurations. We calculated energy differences along each of the different spin orientations within the collinear approximation to the magnetic ground state, but were unable to extract self consistent anisotropy energies. Thus, we chose to pursue collinear DFT calculations in the main text and have included an example calculation in the supporting information. Collinear and non-collinear DFT calculations required different sets of parameters. The PBE functional lacks non-collinear physics[7]; thus, relativistic non-collinear calculations including spin-orbit coupling benefit from the use of the LDA exchange-correlation functional with SOC19 norm-conserving pseudopotentials instead. The incompatibility of Hubbard U with spin-orbit coupling in the current version of CASTEP, 23.1, prevented its use in the non-collinear calculations. Nevertheless, the relativistic interactions covered by non-collinear DFT cover the essential physics of the magnetic systems. The LDA refined structures for non-collinear calculations are reported in Tab. S8.

Table S8: Calculated lattice parameters from non-collinear LDA DFT.

	$FeCl_2$(pym)	$FeCl_2$(btd–d_4)	$NiCl_2$(pym)	$NiCl_2$(btd–d_4)
a (Å)	3.33	3.42	3.39	3.46
b (Å)	11.82	12.49	11.52	12.22
c (Å)	6.90	8.58	6.85	8.41
α (°)	89.79	90.00	90.00	90.00
β (°)	90.62	92.85	90.00	91.69
γ (°)	90.31	90.00	90.00	90.00
V (Å^3)	271.66	365.33	267.89	355.18
Bond lengths, r (Å)				
M-Cl1	2.41	2.39	2.36	2.35
M-Cl2	2.40	2.40	2.36	2.36
M-N	2.09	2.07	1.97	1.99

Spin vectors were obtained using supercells of the refined non-collinear LDA structures corresponding to their experimental magnetic ground state. The corresponding figures visualised by C2X are shown in Fig. S31. The magnetic moments arising around each of the metal ions have been integrated and represented using arrows.

We observed that the optimisation of spin vectors in non-collinear relativistic DFT did not drastically alter the direction of spin from its initialised conditions. This allowed several local minima to exist wherein each had spin vectors pointing in different directions. One possible way to find the magnetic ground state configuration of our system would be to span the combination space of possible directions for spin vectors in the expected size of the magnetic supercell. However, given that the expected size of this supercell would be a $2 \times 1 \times 2$ supercell with eight metal atoms, we find the collinear DFT to be sufficient to make our claims in this paper.

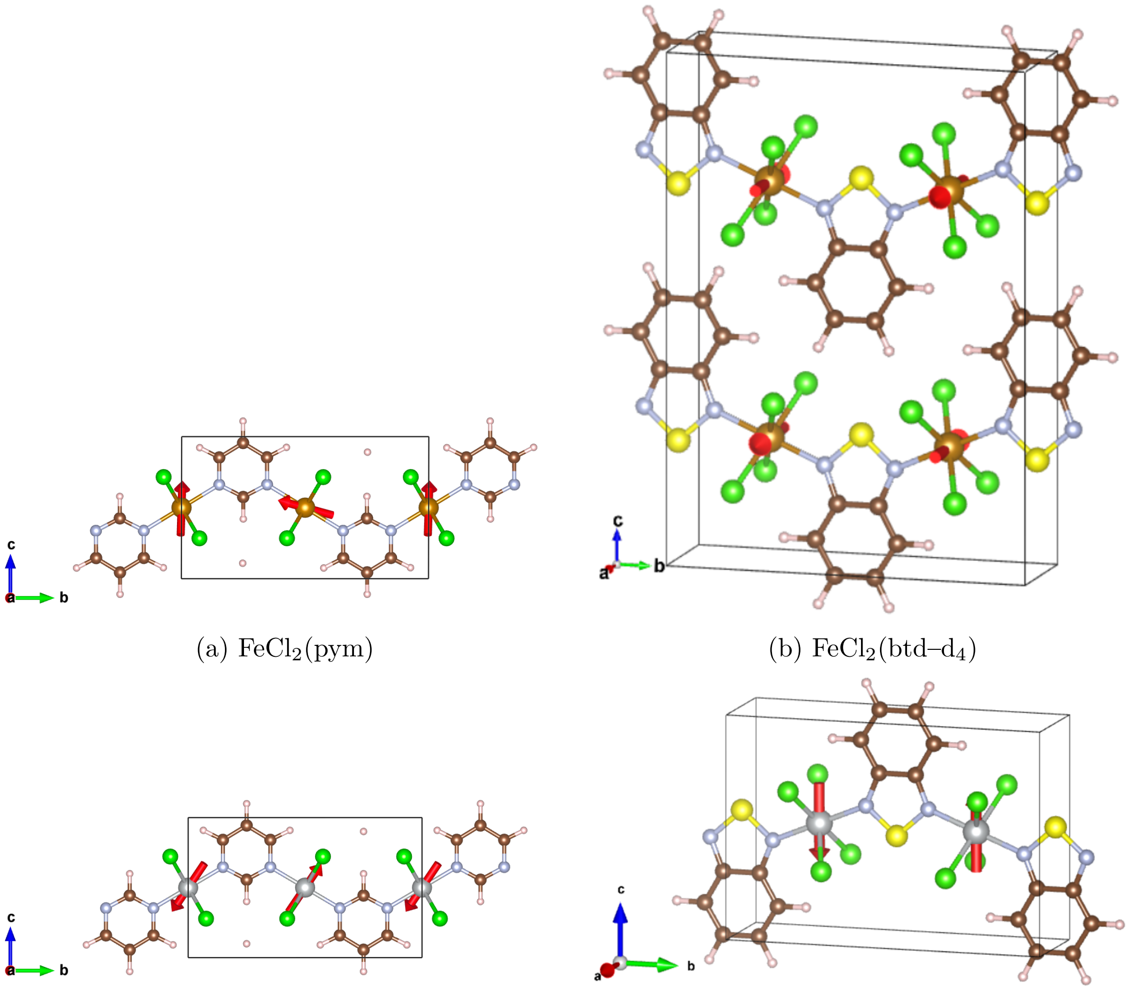

(a) $FeCl_2$(pym)

(b) $FeCl_2$(btd–d_4)

(c) $NiCl_2$(pym)

(d) $NiCl_2$(btd–d_4)

Figure S31: Non-collinear spin directions on ground state MCl_2L.

References

[1] Mohn, P.; Wohlfarth, E. P. The Curie Temperature of the Ferromagnetic Transition Metals and Their Compounds. *Journal of Physics F: Metal Physics* **1987**, *17*, 2421–2430.

[2] Crangle, J.; Goodman, G. M.; Sucksmith, W. The Magnetization of Pure Iron and Nickel. *Proceedings of the Royal Society of London. A. Mathematical and Physical Sciences* **1971**, *321*, 477–491.

[3] Chen, Q.; Adeniran, O.; Liu, Z.-F.; Zhang, Z.; Awaga, K. Graphite-like Charge Storage Mechanism in a 2D π-d Conjugated Metal–Organic Framework Revealed by Stepwise Magnetic Monitoring. *Journal of the American Chemical Society* **2023**, *145*, 1062–1071.

[4] Perlepe, P. et al. From an Antiferromagnetic Insulator to a Strongly Correlated Metal in Square-Lattice $MCl_2(Pyrazine)_2$ Coordination Solids. *Nature Communications* **2022**, *13*, 5766.

[5] Campbell, B. J.; Stokes, H. T.; Tanner, D. E.; Hatch, D. M. ISODISPLACE: A Web-Based Tool for Exploring Structural Distortions. *Journal of Applied Crystallography* **2006**, *39*, 607–614.

[6] Coelho, A. A. TOPAS and TOPAS-Academic: An Optimization Program Integrating Computer Algebra and Crystallographic Objects Written in C++. *Journal of Applied Crystallography* **2018**, *51*, 210–218.

[7] Bulik, I. W.; Scalmani, G.; Frisch, M. J.; Scuseria, G. E. Noncollinear Density Functional Theory Having Proper Invariance and Local Torque Properties. *Physical Review B* **2013**, *87*, 035117.
