# Supplementary material for: Controlling Noncollinear Ferromagnetism in van der Waals Metal–Organic Magnets
Source: J Am Chem Soc. 2024 Jul 2;146(28):19146–59. doi: 10.1021/jacs.4c04102 (PMC11258693; doi:10.1021/jacs.4c04102)

checkCIF/PLATON report

Structure factors have been supplied for datablock(s) fejpla_pmmb

THIS REPORT IS FOR GUIDANCE ONLY. IF USED AS PART OF A REVIEW PROCEDURE FOR PUBLICATION, IT SHOULD NOT REPLACE THE EXPERTISE OF AN EXPERIENCED CRYSTALLOGRAPHIC REFEREE.

No syntax errors found. CIF dictionary Interpreting this report

Datablock: fejpla_pmmb

Bond precision: C-C = 0.0080 A Wavelength=0.71075

Cell:	a=3.5644(2)	b=12.2995(3)	c=7.0800(4)
	alpha=90	beta=90	gamma=90
Temperature:	120 K		

	Calculated	Reported
Volume	310.39(3)	310.39(3)
Space group	P m m b	P m m b
Hall group	-P 2b 2	-P 2b 2
Moiety formula	C4 H4 Cl2 Fe N2	C4 H4 Cl2 Fe N2
Sum formula	C4 H4 Cl2 Fe N2	C4 H4 Cl2 Fe N2
Mr	206.84	206.84
Dx,g cm-3	2.213	2.213
Z	2	2
Mu (mm-1)	3.179	3.179
F000	204.0	204.0
F000'	205.30	
h,k,lmax	4,15,8	4,15,8
Nref	379	379
Tmin,Tmax	0.855,0.900	0.239,0.508
Tmin'	0.735	

Correction method= # Reported T Limits: Tmin=0.239 Tmax=0.508
AbsCorr = GAUSSIAN

Data completeness= 1.000 Theta(max)= 26.060

R(reflections)= 0.0398(368) wR2(reflections)= 0.1045(379)

S = 1.313 Npar= 31

The following ALERTS were generated. Each ALERT has the format
test-name_ALERT_alert-type_alert-level.
Click on the hyperlinks for more details of the test.

Alert level C

```
PLAT250_ALERT_2_C Large U3/U1 Ratio for <U(i,j)> Tensor(Resd    1)        2.6 Note
PLAT341_ALERT_3_C Low Bond Precision on  C-C Bonds ..............      0.008 Ang.
PLAT906_ALERT_3_C Large K Value in the Analysis of Variance ......      3.876 Check
```

Alert level G

```
PLAT004_ALERT_5_G Polymeric Structure Found with Maximum Dimension          2 Info
PLAT128_ALERT_4_G Alternate Setting for Input Space Group  Pmmb          Pmma Note
PLAT794_ALERT_5_G Tentative Bond Valency for Fe1       (II)      .       1.88 Info
PLAT804_ALERT_5_G Number of ARU-Code Packing Problem(s) in PLATON           4 Info
PLAT969_ALERT_5_G The 'Henn et al.' R-Factor-gap value ..........       6.13 Note
              Predicted wR2: Based on SigI**2  1.71 or SHELX Weight  8.31
PLAT978_ALERT_2_G Number C-C Bonds with Positive Residual Density.          1 Info
```

0 **ALERT level A** = Most likely a serious problem - resolve or explain
0 **ALERT level B** = A potentially serious problem, consider carefully
3 **ALERT level C** = Check. Ensure it is not caused by an omission or oversight
6 **ALERT level G** = General information/check it is not something unexpected

0 ALERT type 1 CIF construction/syntax error, inconsistent or missing data
2 ALERT type 2 Indicator that the structure model may be wrong or deficient
2 ALERT type 3 Indicator that the structure quality may be low
1 ALERT type 4 Improvement, methodology, query or suggestion
4 ALERT type 5 Informative message, check

It is advisable to attempt to resolve as many as possible of the alerts in all categories. Often the minor alerts point to easily fixed oversights, errors and omissions in your CIF or refinement strategy, so attention to these fine details can be worthwhile. In order to resolve some of the more serious problems it may be necessary to carry out additional measurements or structure refinements. However, the purpose of your study may justify the reported deviations and the more serious of these should normally be commented upon in the discussion or experimental section of a paper or in the "special_details" fields of the CIF. checkCIF was carefully designed to identify outliers and unusual parameters, but every test has its limitations and alerts that are not important in a particular case may appear. Conversely, the absence of alerts does not guarantee there are no aspects of the results needing attention. It is up to the individual to critically assess their own results and, if necessary, seek expert advice.

Publication of your CIF in IUCr journals

A basic structural check has been run on your CIF. These basic checks will be run on all CIFs submitted for publication in IUCr journals (*Acta Crystallographica*, *Journal of Applied Crystallography*, *Journal of Synchrotron Radiation*); however, if you intend to submit to *Acta Crystallographica Section C* or *E* or *IUCrData*, you should make sure that full publication checks are run on the final version of your CIF prior to submission.

Publication of your CIF in other journals

Please refer to the *Notes for Authors* of the relevant journal for any special instructions relating to CIF submission.

PLATON version of 06/01/2024; check.def file version of 05/01/2024

Datablock fejpla_pmmb - ellipsoid plot

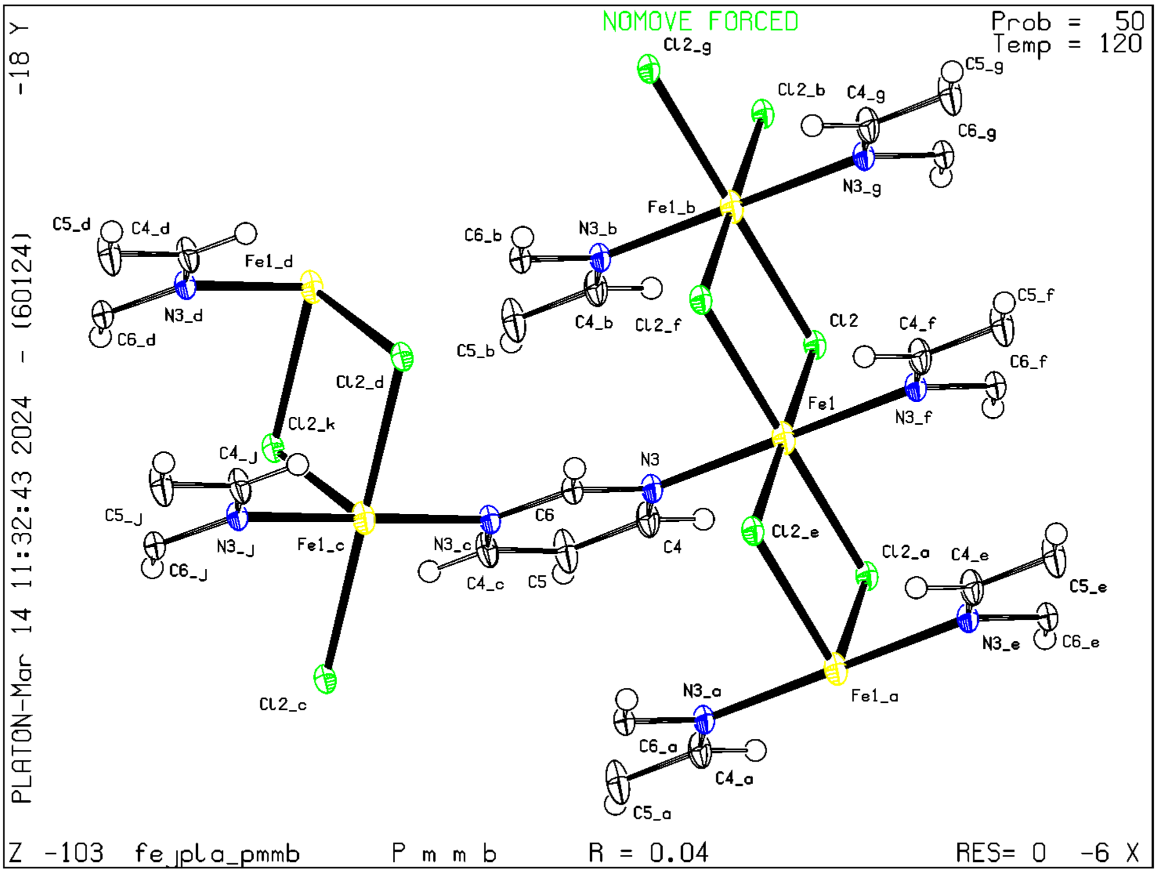

Supplement: Supplementary file 1 — ja4c04102_si_001.zip [file ja4c04102_si_001.zip › CIFs.zip!/CIFs/FeCl2pym_SXD_120K_cifreport.pdf]
